# Seismic characterization of lava flow facies in the critical zone of the deccan traps using shear wave velocity models

**DOI:** 10.1038/s41598-025-13638-4

**Published:** 2025-08-01

**Authors:** Rashi Sharma, Rahul Dehiya, Sudipta Sarkar, Raymond Duraiswami

**Affiliations:** 1 Department of Earth and Climate Science, Indian Institute of Science Education and Research Pune, Pune, 411008 India; 2 Department of Geology, Savitribai Phule Pune University, Pune, 411007 India

**Keywords:** Critical zone, Deccan volcanic province, shear-wave velocity of basalt, MASW, Geology, Geophysics, Volcanology

## Abstract

The critical zone is the uppermost layer of Earth’s crust, where the geosphere, hydrosphere, atmosphere, and biosphere interact to sustain life. In continental flood basalt provinces, its structure and evolution remain poorly understood due to lithological complexities and variable weathering patterns. Geological and geophysical characterization of the subsurface is essential to unravel these factors. Despite advances in understanding basalt lava flow stratigraphy in the Deccan Volcanic Province (DVP), field-scale seismic velocity variations within these flows and their internal structure remain largely unknown. This study integrates seismic data with volcanological information to investigate weathering patterns in the uppermost 50 m of basalt lava flows around Pune city in the western DVP. Using the multi-channel analysis of surface waves technique, we estimate shear wave velocity variations across flow units and dykes. By co-analyzing seismic data with morphological variations across outcrops, we develop a field-scale velocity characterization across basalt lavas and dykes. Critical zone facies, identified and validated through outcrop studies, include soil, weathered bedrock with vesicular basalt, columnar-jointed lava cores, red bole, and intrusive dykes. An analysis of vegetation distribution, landscape morphology, and lithological variability provides insights into key weathering and erosional processes shaping the critical zone in this volcanic terrain.

## Introduction

The critical zone is the outermost layer of the Earth’s surface that comprises the soil, saprolite and the unaltered bedrock^1,2^. The interaction among the topmost part of the lithosphere with the atmosphere, hydrosphere and biosphere drives physical, chemical and biological weathering that enables the transformation of rocks into soil^3^. The zone regulates hydrological and biogeochemical cycles and supports ecosystem and all forms of life^4–7^. The porosity and permeability variations in this zone affect surface water infiltration, water-holding capacity and release of life-supporting nutrients that maintain biological productivity^8^. Lithological and structural variations such as fractures, joints, formed under tectonic stress along with climatic factors such as temperature, rainfall variability contribute to the development of the critical zone. These factors ultimately control landscape evolution, hydrologeological conditions at a local and regional scale^9,10^.

Geophysical methods have proven effective in examining a wide range of aspects within critical zone research. These include quantifying deformation (volumetric strain) below hillslopes^19^, estimating water storage capacity^16,20,21^, and exploring the links between the critical zone and drought resilience^22–24^. A multiscale geophysical approach is most desired for comprehensive characterization, as it integrates various geophysical methods to analyze subsurface properties^25^. For example, active-source electromagnetic methods are sensitive to electrical conductivity and enable deep signal penetration, though with lower spatial resolution, while Nuclear Magnetic Resonance can directly sense the water content of the medium. Electrical Resistivity Tomography and self-potential methods help detect fluid flow and electrochemical transport processes. Additionally, satellite-based gravity data, such as from the Gravity Recovery and Climate Experiment (GRACE), provide continental-scale insights into geological structures and hydrologic storage changes.

Among these techniques, seismic methods are widely applied to determine the velocity structure and mechanical properties of the critical zone. Active-source seismic reflection and refraction surveys using sledgehammers, weight drops, or explosives generate body and surface waves to image the subsurface^15^. Seismic refraction of compressional waves (P-waves) has become a common tool for delineating subsurface velocity structures^11–14^. P-wave velocity models derived from field surveys ($\sim$ 50 m scale) and sonic logs ($\sim$ 0.5 m scale) help reconstruct the broad structural framework and reveal lateral and vertical heterogeneities^16,17^. The Poisson’s ratio further assists in identifying zones with fractured bedrock and saprolite^18^. In this study, we adopt seismic methods to investigate the spatial variability of weathering and fracture zones in basalt-dominated critical zone environments.

Continental flood basalts (CFB) develop distinct soil profiles and extensively altered basalt when exposed to intense chemical and physical weathering. In rapidly urbanizing cities like Pune, Nagpur and Mumbai etc. on basaltic terrain, the characteristics of near-subsurface geological formations play an important role in groundwater availability and slope stability. Understanding heterogeneity is critical for accurate groundwater reservoir estimation, mechanical strength assessment, and identification of the geological controls that influence them. As engineering projects related to urban development alter the natural landscape, it becomes critical to assess the stability of subsurface geological layers to ensure infrastructure safety and sustainable development. Since seismic methods serve as an effective tool to image the subsurface, it is critical to understand the relation between subsurface physical characteristics and seismic velocities ($V_P$, $V_S$) to determine the suitability of seismic methods in characterizing the internal heterogeneity of the critical zone structure and basaltic bedrocks (e.g.,^25^). In CFBs, basalt lava flows exhibit significant lateral and vertical lithological and structural heterogeneities, including variations in joint density and interflow weathered horizons, which can have substantially lower velocities compared to the massive basalt layers. This heterogeneity within the basalt sequence influences both its mechanical behaviour and seismic response. The presence of low-velocity zones does not allow the critical refraction to take place from the low velocity layer making it difficult to image using refraction tomography. An alternative approach could be to apply the Multichannel Analysis of Surface Waves (MASW) technique that uses Rayleigh wave dispersion curves to estimate $V_S$ models of the subsurface^26^. Additionally, integrated geological and geophysical analyses of basalt lavas can provide analogue models to enhance geophysical data interpretation and develop synthetic models for hydrocarbon exploration in CFBs. More applications lie in their potential for carbon capture and storage^27^.

The objective of this study is to conduct near-surface seismic characterization of lava flows intruded by dykes within a large CFB like the Deccan Traps, to better understand variations within the critical zone and the geological complexities associated with the bedrock that reflect complex weathering patterns of basalts. We addressed this by implementing the MASW technique using a short array to derive S-wave velocity models that help characterize the structure and variability of the weathered near-surface profile and the underlying geological features associated with basalt lava flows. We also correlate the MASW-based S-wave velocity models with geological information from field outcrops to support our interpretations.

## Geological background

**Fig. 1:**
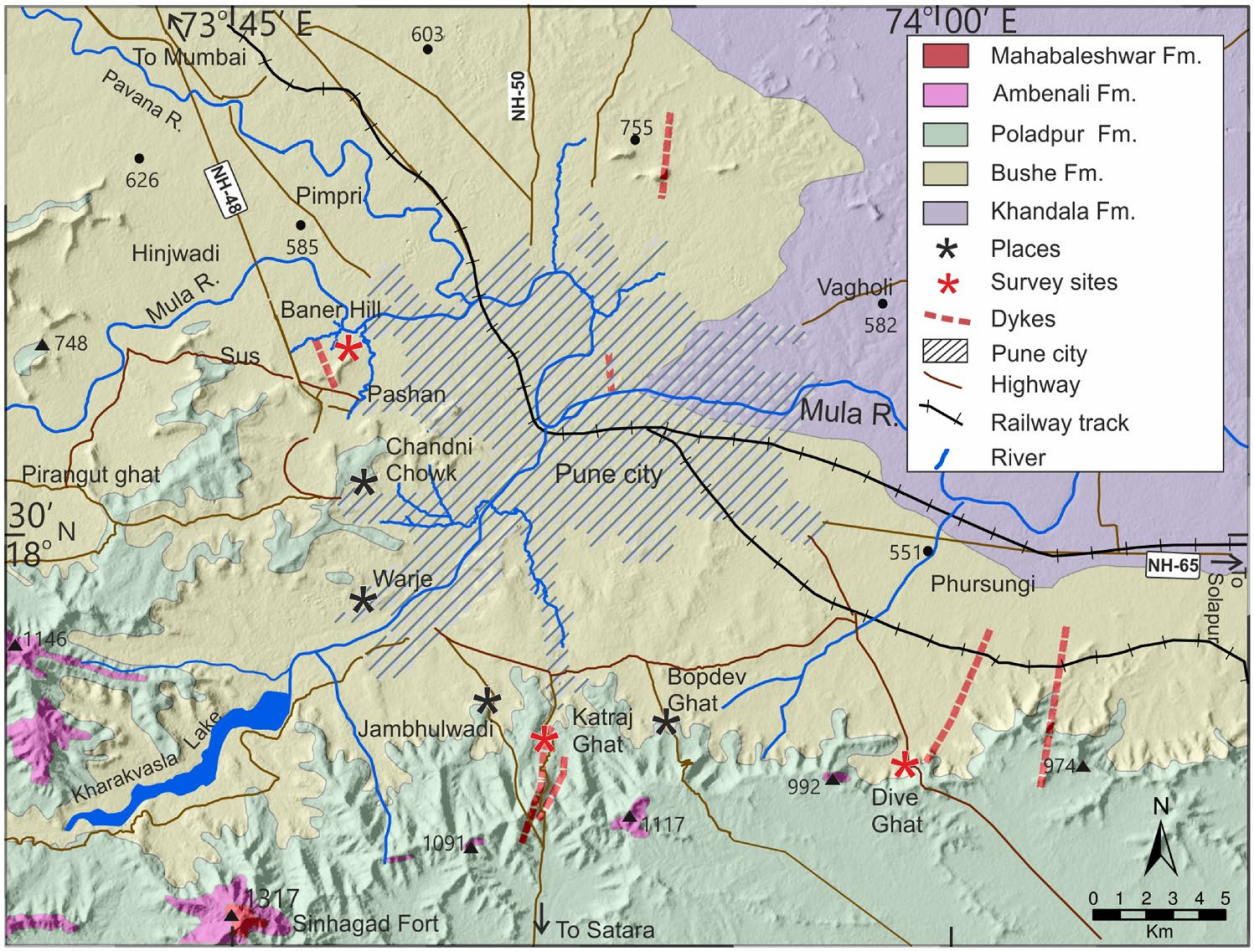
Geological map of the Pune region showing the diverse Formations in the study area. Geological Formations are taken from^36^.

The Deccan flood basalt (Cretaceous–Paleogene) covers an area of $\sim$5,00,000 km$\phantom{0}^2$ and the entire basalt lava sequence has been stratigraphically divided into three major subgroups and thirteen formations^28,29^. The region surrounding Pune (Fig. 1) is characterized by exposures of basaltic lava flows belonging to the Deccan Traps^29,30^. In the Yerwada-Katraj Ghat section, 25 distinct lava flows have been identified^31^, with thicknesses ranging from approximately 2.5 to 40 m. These flows exhibit diverse morphologies, including hummocky pahoehoe, sheet pahoehoe, rubbly pahoehoe, and 'a'ā types^32^. Pahoehoe lobes and sheets are characterized by a vesicular crust, a massive core, and a pipe-bearing basal zone^32^. The rubbly pahoehoe flows are characterized by a thick flow-top breccia^33,34^, whereas 'a'ā flows exhibit both flow-top and flow-bottom breccias^35^. The vesicular crust (vesicular basalt) typically has high porosity or is infilled with hydrous zeolites (amygdaloidal basalt). Jointing within the massive core may further influence porosity, thereby affecting the petrophysical properties. The flows in the Pune region are assigned to the Khandala Formation, followed by the Bushe Formation, Poladpur Formation, and Ambenali Formation. The sequence is intruded by a north-south-trending swarm of five dykes (Fig. 1), ranging in width from 0.3 to 8.9 m. A brief overview of the basalt facies observed in the study area is presented in Table 1 (also refer to Supplementary Text S1.1.).

Lava flows are often separated by interflow red, brown, or green ‘bole’ horizons, which form due to in situ weathering of basalt flow tops during the periods of eruptive quiescence^35^. Their distribution is uneven, with boles being more prevalent in the Pune region and increasingly abundant in the younger formations of the Wai Sub-group^37^. Although pervasively weathered, boles can also appear laminated or structureless and indurated due to baking by subsequent lava flows^38^.

**Table 1:** Different basalt facies with their brief description. Field photographs and their schematics are of the same scale.

Field Photograph	Line drawing illustrating the key features	Facies Name	Description
		Sheet pahoehoe	Sheet pahoehoe is a type of pahoehoe lava flow that is characterized by laterally extensive, and continuous lava sheets. This structure consists of three distinct units-the vesicular crust, the flow core, and a basal pipe-bearing zone. The core often shows columnar jointing and spheroidal weathering is seen near the surface.
		Hummocky pahoehoe	A pahoehoe lava consisting of lobes. The top surface of this type of lava is smooth and bun-like. Inverted-Y shaped vesicles are seen near the base of the lobe.
		Indurated Red Bole	A compact form of red bole, often showing a well-lithified/structured appearance.
		Red bole with lava ball	A red bole facies containing large clasts or rubble balls embedded within the fine-grained matrix.
		Onion skin weathering in Red Bole	Characterized by concentric weathering patterns, resembling onion skin-like peeling.
		Rubble Red Bole	A highly fragmented and brecciated variety, often found between lava flows.
		Basaltic Dykes	Sheet-like intrusion and discordant to the country rocks and shows internal cooling joints
		Vesicular/amygdular basalt	Lava flow characterized by the presence of vesicles, which may or may not be filled with secondary minerals.
		Massive (Jointed) Basalt	Basalt characterized by a dense, non-porous texture that often develops joints due to cooling.

The origin and evolution of bole beds in the Deccan basalts remain highly debated^39^. Some authors^37,40^ proposed that bole layers are weathered pyroclastic deposits representing fossilized paleosurfaces, while others^41–43^ view them as paleosols. The red boles owe their colour to varying amounts of Fe$\phantom{0}^2$O$\phantom{0}^3$ and are dominated by smectite, a product of chemical weathering of basalt through interaction with meteoric water^41^. Smectite forms in tropical and subtropical regions where alternating wet and dry seasons, flat terrain, and poor drainage promote pedogenesis^44^. During wet periods, rising water table and the reducing conditions mobilize iron as Fe$\phantom{0}^{2+}$, while drying causes iron to precipitate within clays and iron-(oxy)hydroxides like goethite or ferrihydrite, eventually developing into a plinthite (brick red layer) horizon^45^. Clay mineralogy reflects bole formation processes^46^. Pedogenic weathering of volcanic or volcaniclastic material produces a chemically heterogeneous mixture of kaolinite and smectite, evident in fragile red boles. Poor drainage and periodic mixing of meteoric water with rainwater influenced smectite and kaolinite formation^46,47^. Saprolitic red bole forms through the pedogenic weathering of basaltic flow tops, and develops chemically diverse assemblage of smectite and kaolinite. The green, and brown boles have been linked to andosolization^41,46^.

## Data and methodology

### Seismic data

**Fig. 2:**
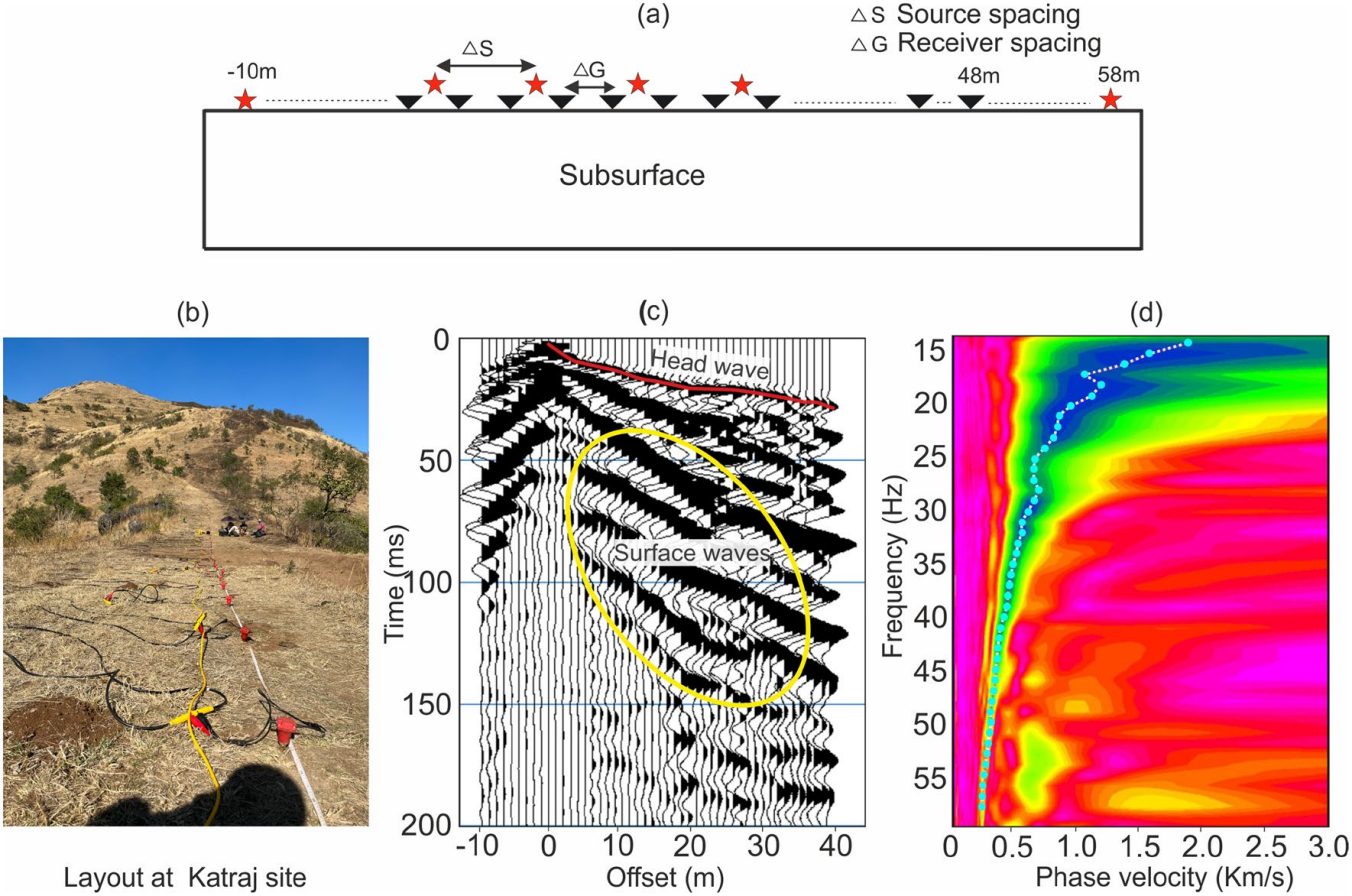
Seismic survey and an example of seismic data. (**a**) Illustration of acquisition geometry. (**b**) Geophones and cables spread over the ground during data acquisition. (**c**) Example of a raw shot-gather from the Katraj profile (KJP1) showing the surface waves and head waves (first breaks). (**d**) Phase velocity frequency data example. The cyan dots were picked corresponding to the fundamental mode of frequency at maximum semblance. The picked dispersion curve was then used in the inversion process.

We acquired seismic data using a 48-channel Geometrics Geode Engineering Seismograph with 4.5 Hz vertical geophones, and a 7 kg-sledgehammer as the energy source to examine the top tens of meters of the subsurface. A schematic diagram representing the survey design is shown in Fig. 2a. Seismic data were collected from five locations in the Pune region (Fig. 1): Baner Hill (1 profile), Dive Ghat (2 profiles) and Katraj (2 profiles). At Baner Hill, seismic data was acquired in 2021 with receiver and source intervals of 3 and 6 m, respectively using a 24-channel engineering seismograph (Geode system). Consequently, the receiver spread length in this survey was 69 m. All other profiles were acquired using a 48-channel engineering seismograph (Geode system) in 2023 and 2024 with the receiver and source intervals of 1 and 2 m, respectively. A field layout and a shot point gather from Katraj site are shown in Figs. 2b and 2c, respectively. The topography along the profiles in all five cases is flat with very minor undulations. Due to the short spread, direct and surface waves obscure the reflection events, restricting the data analysis to the surface waves. Therefore, conventional reflection analysis could not be conducted with this data.

### Velocity estimation

We estimated two-dimensional (2D) subsurface S-wave velocity using the MASW technique^15^. We first generated common midpoint gathers (CMP) by cross-correlating recorded seismic traces and combining them within similar offset ranges. These cross-correlated CMP gathers are referred to as CMPCC. Near-offset traces (< 10 m offset) were excluded from the CMPCC computation. Each CMPCC gather was then transformed from the time-offset domain (Fig. 2c) to the frequency-phase velocity (Fig. 2d) domain. The CMPCC bin size was set to twice the receiver interval, resulting in a bin size of 6 m at Baner Hill and 2 m at the other sites. The frequency range suitable for inversion depends on the spread length, source characteristics and receiver frequency. In this study, a usable frequency range was identified to be approximately 15 to 60 Hz (Fig. 2d). The phase velocity of the fundamental mode was picked on each CMPCC gather, and the frequency-phase velocity curves were obtained. Each CMPCC gather was modelled using a 1D algorithm that simulates phase velocity for a given frequency, considering that velocity varies with depth beneath the midpoint. Next, we estimated 2D $V_S$ variations by simultaneously analyzing all frequency-phase velocity curves using the inverse modelling technique. This workflow, extensively applied in previous research studies^48–52^, has proven very effective in deriving 2D S-wave velocity distributions.

### Inversion scheme

Inverse modelling mentioned above strives to find a model that fits the observed data within a tolerance. The tolerance is governed by the noise in the data and the numerical accuracy of the modelling algorithm used in the inversion code. Data misfit is commonly defined in a least-square sense. Since inverse modelling is an ill-posed problem leading to instability, it is stabilized using a regularization technique. The regularization operators can be seen as the inverse of the model covariance matrix^53^. Two types of regularization operators were employed in the inversion algorithm, (i) first-order derivative in the direction of depth and (ii) depth-dependent weighting. The first-order derivative in depth generates a vertically smooth velocity estimate. In the case of the second operator, the weights increase with depth, and the matrix is diagonal. As a result, the variance for deeper subsurface velocities is reduced because the model covariance is the inverse of the regularization operator. Consequently, the bias due to the second operator increases with depth. We did not apply any horizontal smoothing, as we focus on sharp vertical/near-vertical variations required for imaging distinct features, such as dykes that cut across the basalt flows. In this study, we have used SeisImager software (Geometrics) for data analysis and pseudo-2D inversion. The inversion process is a non-linear problem, where the model is iteratively updated until no significant changes are observed, at which point the final model is accepted. The initial model is vertically divided into 50 cells. The thickness of the first layer is 0.5 m and increases linearly with depth at a rate of 0.03 m per cell for KJP1 profile and 0.02 m per cell for the other profiles. The horizontal cell size matches the CMPCC bin size. A regularization factor of 0.5 is used, and no horizontal smoothing is applied. Velocities were assigned using the frequency-phase velocity curves, and the model was iteratively updated until convergence. The uncertainty in the estimated subsurface velocities, expressed as ${\textbf {C}}_{pm}$, was calculated for linear inverse problems assuming Gaussian statistics, following

1 $$\begin{aligned} {\textbf {C}}_{pm} = \left( {\textbf {J}}^T {\textbf {C}}_d^{-1} {\textbf {J}} + {\textbf {C}}_m^{-1}\right) ^{-1} \end{aligned}$$

where ${\textbf {C}}_d$ and ${\textbf {C}}_m$ represent the data and model covariance matrices respectively and **J** is the Jacobian matrix^53^. A key challenge in uncertainty estimation is the impact of the regularization operator, particularly when model variance is low, leading to substantial bias. In such cases, the estimated model is constrained by prior information rather than by the data.

The uncertainties were computed for non-linear problems using Equation 1 in the neighbourhood of the final inverted model. This required a data covariance matrix, which in this study, was assumed to have independent errors, meaning only diagonal terms were non-zero. Additionally, data errors were considered to have an identical standard deviation. Since the inversion algorithm was based on 1D modelling, data errors arose from two primary sources- errors in observed data and the errors due to 1D approximation. The inversion was allowed to converge and the residual misfit was considered as an error, indicating the inability of the algorithm to fully fit the data. This cumulative error was assumed to follow a normal distribution, implying that data errors were Gaussian. A normal distribution was then fitted to the misfit to determine the standard deviation, which was then used for uncertainty estimation.

The final shear velocity models for all the profiles are shown in Figs. 6-10. The misfit between the picked phase velocity data and the simulated data from the final model was computed to estimate data error. The standard deviation of the misfit for each profile was computed by fitting a normal distribution through trial-and-error approach. Finally, the uncertainties in the estimated shear velocity model were calculated, with the data variance obtained for the corresponding data misfit.

## Results

**Fig. 3:**
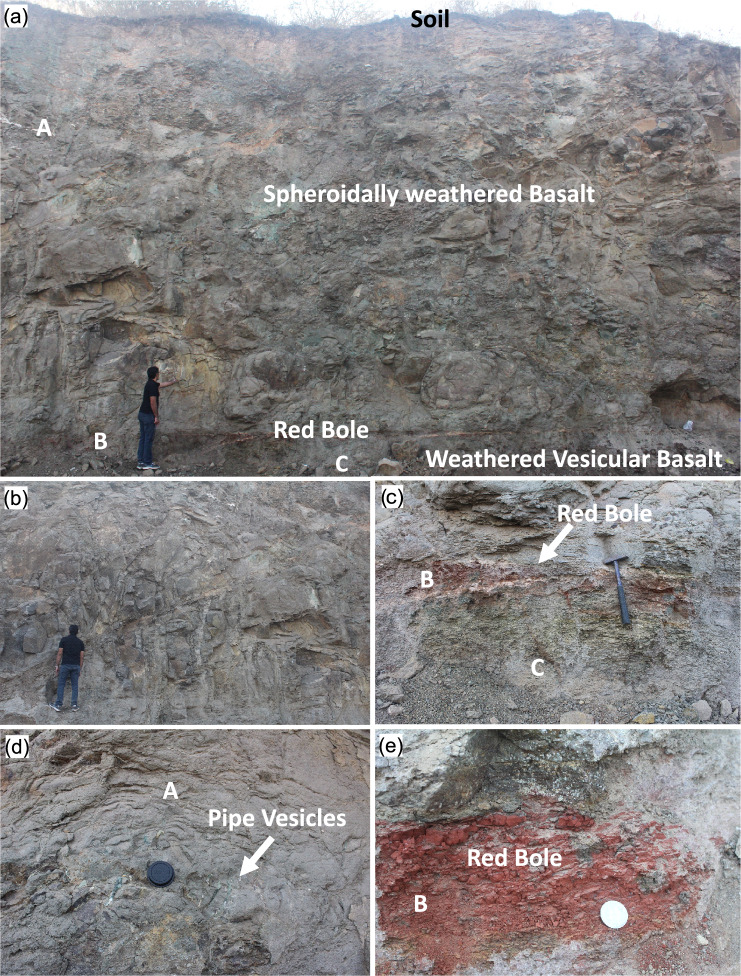
Field photographs of a geological section at the Dive Ghat site, taken parallel to the survey profile DGP1, with white-labelled markers (A, B, and C) indicating key lithological units: spheroidally weathered basalt, red bole, and weathered vesicular basalt, respectively. (**a**) A broader view of the 11 m vertical section, showcasing the spheroidally weathered basalt, red bole, and the upper part of the weathered vesicular basalt. (**b**) Cooling and columnar joints within the spheroidally weathered basalt. (**c**) The red bole layer of varying thickness, positioned between the spheroidally weathered basalt and the weathered vesicular basalt. (**d**) Pipe vesicles filled with greenish secondary minerals at the base of the spheroidally weathered basalt, marking the base of the lava flow. (**e**) A close-up view of the red bole, highlighting its internal structure.

**Fig. 4:**
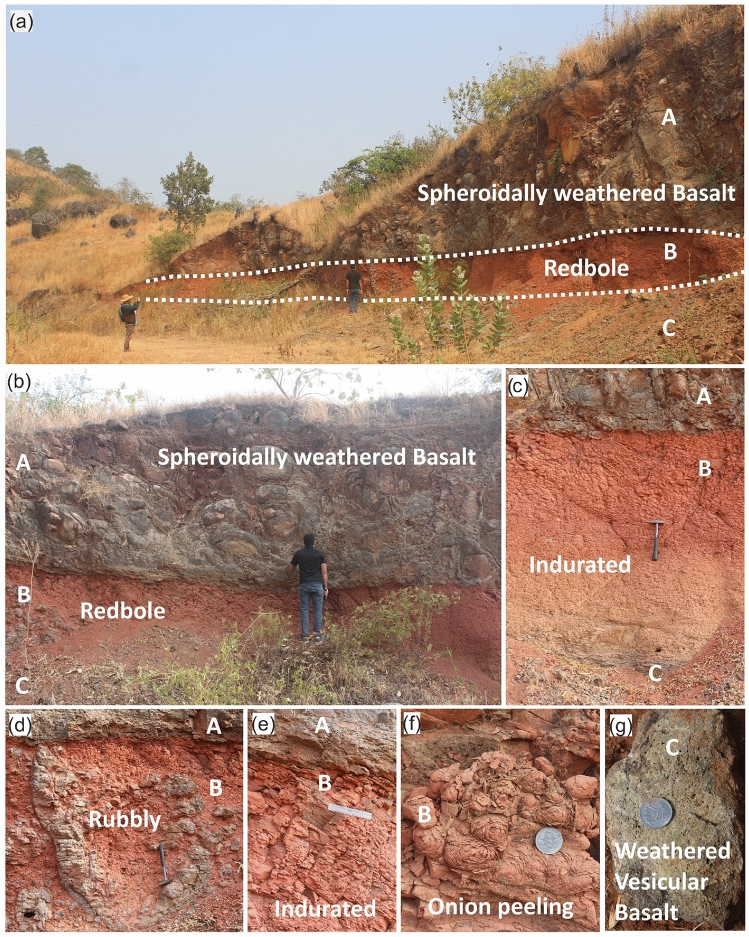
Field photographs of the Katraj section along the survey profile KJP1, with white-labelled markers (A, B, and C) indicating the spheroidally weathered basalt unit, red bole, and weathered vesicular basalt, respectively. (**a**) A side-view perspective of the 7.5 m vertical section, showing the spheroidally weathered basalt, red bole, and the uppermost part of the weathered vesicular basalt. (**b**) A close-up view highlighting the internal structure of the spheroidally weathered basalt and its contact with the red bole. (**c**) The red bole layer, showcasing its indurated structure. (**d**) A lava ball approximately 1.5 m in diameter embedded within the red bole; (**e**) a detailed view of the red bole, revealing its indurated, dipping structure relative to its contact with the spheroidally weathered basalt. (**f**) An onion-peeling structure within the red bole, illustrating spheroidal weathering at the centimeter scale. (**g**) A sample of weathered vesicular basalt extracted from approximately 0.25 m below the red bole base.

**Fig. 5:**
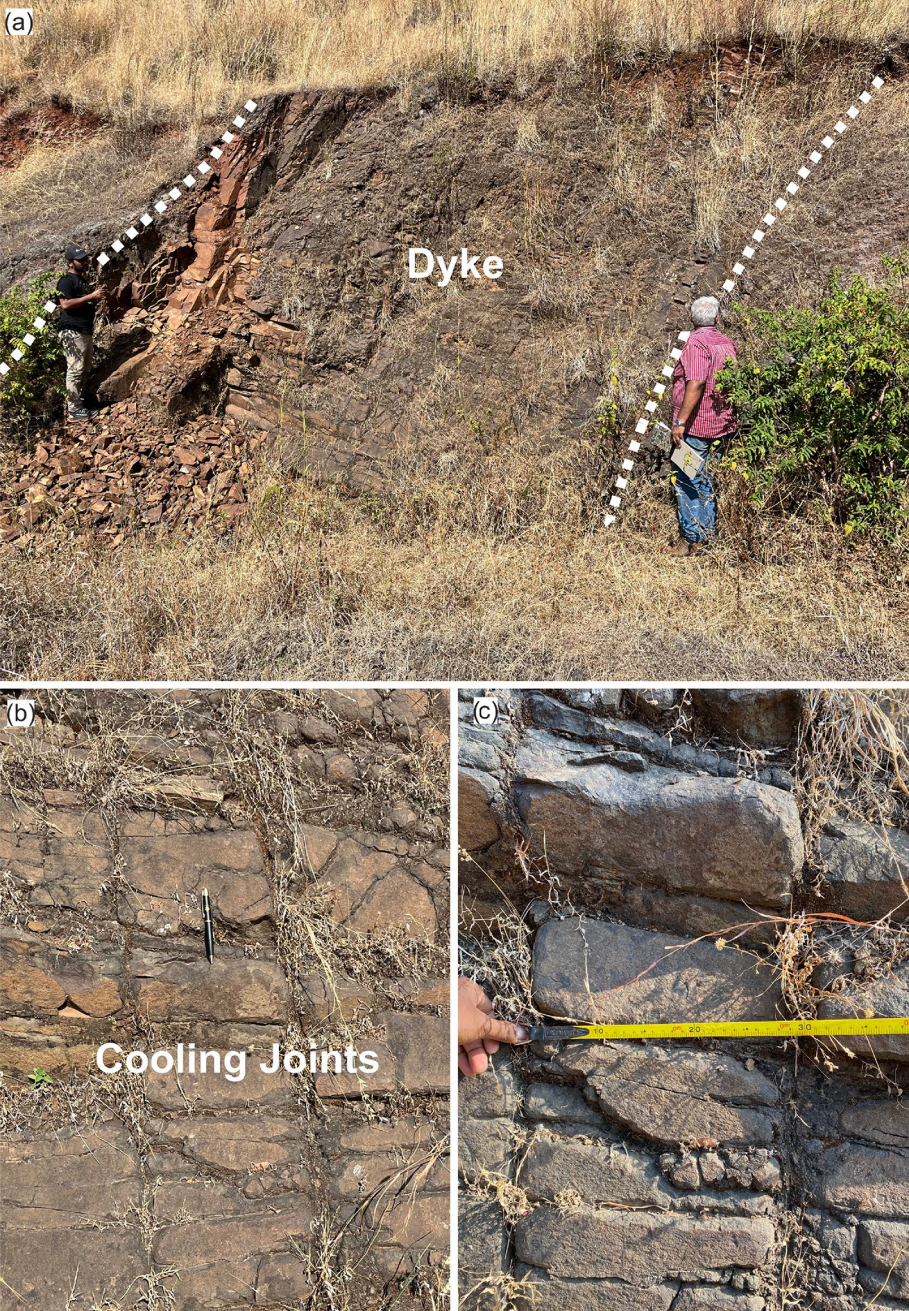
Field photographs of the Katraj dyke, taken adjacent to the survey profile KJP2, highlighting its width and cooling joints. (**a**) A view of the dipping dyke, with dashed white lines marking its boundaries, indicating its inclined nature, while the cooling joints are oriented perpendicular to these boundaries helping in delineating the margins of the dyke. (**b**) A close-up view of the dyke, showcasing the characteristics of the cooling joints. (**c**) An image illustrating the spacing of the cooling joints, which are approximately 30 cm apart horizontally and 10 cm apart vertically.

In the following sections, we first briefly describe the field-scale geological features related to the basalt flows observed in the accessible and exposed outcrops at each seismic profiling site (Refer to section S1.2. in Supplementary document). We then present the seismic profiling results, which characterize subsurface velocity variations and uncertainties, and correlate these findings with outcrop descriptions. The geological features at various scales of the survey area are illustrated in the field images as described in Figs. 3, 4 and 5. Petrographic analysis was conducted in various samples from the study area, with the results summarised in Supplementary Text S1.3. and Supplementary Figure S5.

The geological sections (schematic representation) for all the sites are also presented for the outcrops based on geological fieldwork and physical volcanological principles.

### Baner Hill (BH) profile

At the Baner Hill site, compound pahoehoe lava flows of the Bushe Formation are exposed, forming small hillocks. These flows display a compound structure, consisting of a stacked sequence of hummocky lobes alternating with tabular sheet lobes. Many of the pahoehoe lobes are vesicular, containing gas blisters and pipes with an inverted Y-shaped morphology near their base. These blisters are often lined with secondary minerals, such as stilbite, apophyllite, and calcite^54^.The presence of tumuli near the uppermost sections further suggests variability in the dynamics of lava inflation^55–57^. Tumuli are formed by lava inflation, which creates clefts that are filled with material pushed upwards, often referred to as “squeeze-ups” (refer to Supplementary Figure S7).

**Fig. 6:**
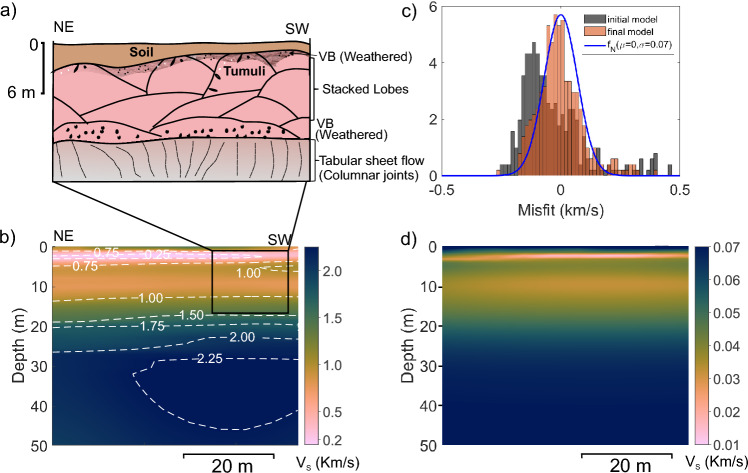
Lava flow layers and $V_s$ model at the Baner Hill Site. (**a**) Schematic diagram illustrating the lava flow geometry in the survey area based on an outcrop exposed in a nearby quarry. Quarry location is shown in Supplementary Figure S1. (**b**) Shear wave velocity profile. (**c**) Histogram representation of data misfit for the first and final inversion iterations along with a normal distribution fitted to the last iteration misfit. (**d**) Uncertainty estimates in the inverted velocity profile. Supplementary Figure S9 shows an overlay image of the velocity model and schematic basalt flow layers for the selected inset.

In the spur separating two hillocks, a basaltic dyke, 3-5 m thick, is exposed. Due to topographic constraints, the seismic array could not be deployed across this dyke. In the western hillock (Fig. 6a), the stacking of 8 m thick pahoehoe lava lobes is visible in the upper flow layers, while a laterally extensive, thick sheet lobe is exposed at the base in a quarry. The upper part of the quarry shows approximately 1-2 m thick vesicular basalt (VB) crust layer showing tumuli, above a massive lava flow core.

Figure 6b illustrates the estimated S-wave velocity model along a 54 m-long profile, extending to a depth of 50 m. The top 2 m of the subsurface exhibits relatively higher velocities (1-1.5 km/s), which is underlain by a thin, low-velocity layer ($\sim$2 m thick with velocity $\sim$0.1 km/s). Between depths of 5 and 8 m, velocities were found to be around 1.0 km/s, followed by a reduction to approximately 0.8 km/s at a depth of 10 m. At around 15 m in depth, the velocity displays a notable increase along with a consistent increasing trend in velocity to a depth of 20 m. Below 25 meters, the velocity exceeds 2.0 km/s, indicating the presence of the lava flow core, with the overlying unit corresponding to the crustal layer of the flow. The schematic representation of the geological section is presented in Fig. 6a and corroborated with the $V_s$ model.

The data misfit corresponding to the first and final inverted model is shown in Fig. 6c. The comparison of the distribution indicated that the inversion has substantially improved the data fit. Furthermore, the misfit distribution of the final model approximates a normal distribution, indicating an unbiased fit. To estimate the standard deviation of data misfit, normal distributions of various variances were tried and a normal distribution of 0.07 standard deviation shown with the blue curve seems to show a reasonable fit to data misfit distribution. The uncertainties in the final inverted model are estimated using a data covariance matrix, assuming a standard deviation of data as 0.07. The uncertainty in velocity is illustrated in Fig. 6d. The maximum uncertainty in the estimated velocity is around 0.07 km/s, which is found in the very shallow and deeper parts of the section. The uncertainties in the shallow part are high because the recorded data lacks the very high frequencies required to resolve the features at the scale of the thinnest topmost cell. It is likely that the uncertainties in the deeper part may be underestimated due to the regularization bias.

### Diveghat (DGP1) profile

**Fig. 7:**
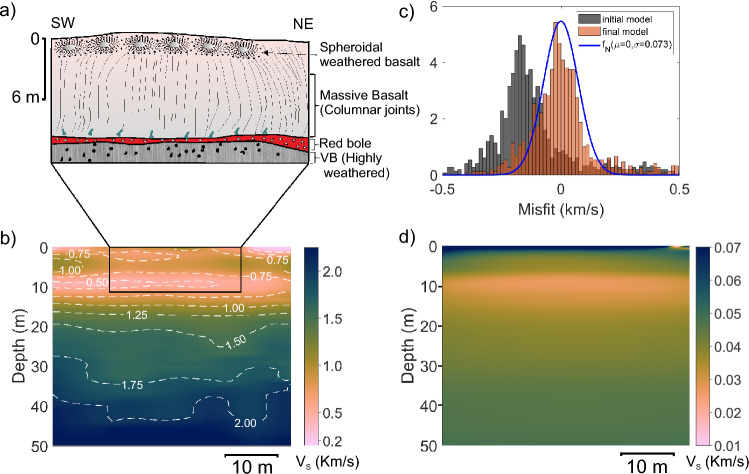
Dive Ghat DGP1 profile results. (**a**) Schematic diagram illustrating the internal structure of lava flow exposed in the survey area based on a section exposed in a nearby quarry, which is parallel to the survey line. Quarry location is shown in Supplementary Figure S2. (**b**) Estimated shear wave velocity profile. (**c**) Histogram representation of data misfit for first and final inversion iterations along with a normal distribution fitted to last iteration misfit. (**d**) Uncertainty estimates in the inverted velocity profile. An overlay image of the velocity model and lava flow layers for the inset is shown in Supplementary Figure S10.

Two lava flows belonging to the Bushe Formation are exposed at the base of the Dive Ghat section (Fig. 7a). The topmost flow is a 9.5 m-thick tabular sheet lobe with a few pipe vesicles at the base and a spheroidal-weathered central core with very few vesicle cylinders. The oxidized crust of this upper flow unit contains spherical vesicles, which crudely define banding. The underlying flow is partially exposed within the quarry. These two lava flows are separated by a highly weathered, interflow red bole horizon. To the east of the quarry section, a flow-top breccia, approximately 1 m thick, is present within a clayey (red bole) matrix. In other parts of the section, a 0.6 m thick red bole layer marks the interflow. The upper part of the underlying flow, just below the red bole layer, consists of highly weathered vesicular basalt. The seismic array was deployed on the uppermost part of the quarry.

The S-wave velocity model derived from inversions for the Dive Ghat section is shown in Fig. 7b. At the top of the section, thin pockets of low-velocity soil layers are present, followed by a velocity increase from 0.5 to 1.2 km/s between 0.5 and 8 m below the surface. This velocity range corresponds to the spheroidally weathered basalt observed in the quarry and labeled by ‘A’ in the field photographs shown in Fig. 3. Between approximately 9 and 12 meters depth, a low velocity of around 0.5 km/s is observed, which correlates with a thin red bole layer and the uppermost part of the weathered vesicular basalt from the lower flow seen in the outcrop and labeled by ‘B’ and ‘C’, respectively, in Fig. 3.

A sharp increase in velocity is observed between 12 and 14 m depth, where the velocity rises from approximately 0.5 km/s to 1.2 km/s. This velocity gradient is likely associated with the transition from the weathered vesicular crust of a lava flow to the underlying core, which is likely to be less weathered than the vesicular surface. Below this depth, the velocity continues to increase with depth up to the base of the model 50 m. It is important to note that the MASW method typically loses resolution with depth due to the prevalence of lower frequencies in deeper sections. As a result, any thin anomalous velocity layers in the deeper parts of the section may not be resolved by this technique.

The data misfit plots for the initial and final model are depicted in Fig. 7c. The misfit for the final model indicates a 0.073 standard deviation, which is used for uncertainty estimation. The calculated uncertainty plot is illustrated in Fig. 7d. The uncertainties are mostly less than 0.03 km/s throughout the profile except in the topmost cells. The higher uncertainties ($\sim$0.06 km/s) in the topmost layer are due to the lack of high frequency. Nonetheless, the overall lower uncertainty values provide confidence in the estimated velocity models.

### Diveghat (DGP2) profile

**Fig. 8:**
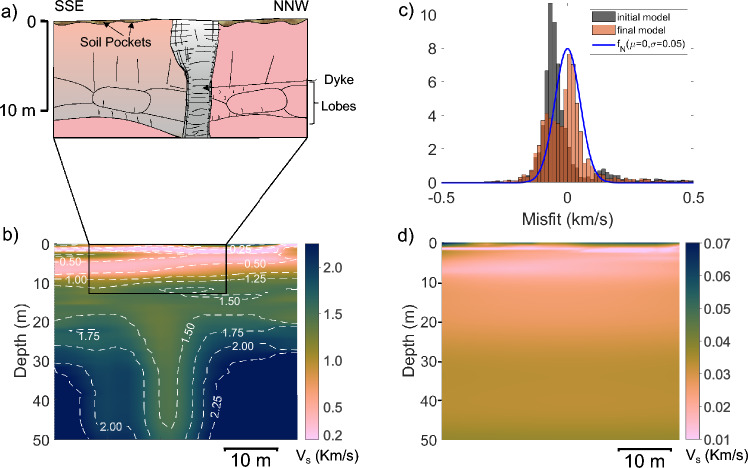
Dive Ghat DGP2 profile results. (**a**) Schematic diagram illustrating the internal structure of lava flow in the survey area based on a section exposed in a nearby area. Quarry location is shown in Supplementary Figure S3. (**b**) Estimated shear wave velocity profile. (**c**) Histogram representation of data misfit for first and final inversion iterations along with a normal distribution fitted to last iteration misfit. (**d**) Uncertainty estimates in the inverted velocity profile. Supplementary Figure S11 shows overlay image of the velocity model and basalt flow layers for the selected inset to facilitate visual comparison.

The pahoehoe lava flows of the Bushe Formation are exposed at the Dive Ghat section (Fig. 1). A doleritic dyke, approximately 2.5-3 m wide, cuts across a topographic ridge near the base of this section (Fig. 8a). The dyke strikes N10$\phantom{0}^o$-S180$\phantom{0}^o$, and intrudes the compound pahoehoe lavas of the Bushe Formation. The dyke exhibits a well-defined chilled margin that is highly jointed in contrast to the relatively intact core. A seismic profile (DGP2) was acquired across the dyke in the SSE–NNW direction.

The inverted S-wave velocity model from the DGP2 profile (Fig. 8b) shows velocities less than 0.5 km/s in the top $\sim$10 m, except at the SSE end, where they locally reach 1.2 km/s. Below 10 m, velocity generally increases with depth, except near the profile centre.

The top 10 m of the section is highly weathered basalt. Below 20 m, a near-vertical low-velocity zone at the profile centre aligns with the location of the dyke. At this location, velocity nearly remains constant at $\sim$1.4 km/s in the depth range from 15-50 m. The velocity anomaly associated with the dyke is not observed in the top 15 m possibly due to weathering, which makes its seismic properties appear similar to the host rock. At greater depths, it exhibits lower velocities than the host flow. The MASW method effectively images the $\sim$3 m thick dyke within the lava flow, with reliable observations up to $\sim$50 m. Figure 8c shows the data misfit plots for the first and final inversion iterations, with the final plot following a normal distribution and a standard deviation of 0.05. The misfit distribution lacks a well-defined bell shape, possibly due to a thin layer of vertical anomaly, which poses challenges for a 1D-inversion algorithm. Further investigation using a true 2D inversion algorithm may resolve this problem. The calculated uncertainties in the S-wave model obtained using data uncertainties with a standard deviation of 0.05 are shown in Fig. 8d. The uncertainties are generally below 0.3 km/s. Interestingly, there is no visible change in the uncertainty estimates related to the dyke and the lava flows. We postulate that it is due to the regularization factor, which influences the estimation of uncertainties. More elaborate uncertainty quantification is possible using a Bayesian approach-based inversion algorithm, which is not attempted in this study.

### Katraj Ghat (KJP1) profile

**Fig. 9:**
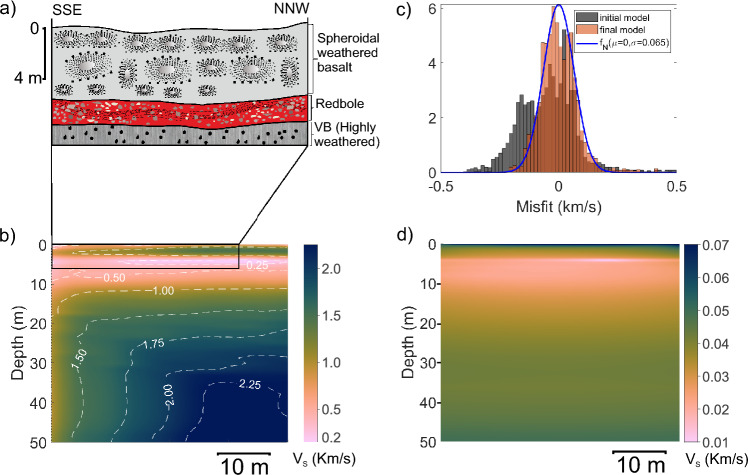
Katraj Ghat KJP1 profile results. (**a**) Schematic diagram illustrating the internal structure of lava flow in the survey area based on a section exposed in a nearby quarry, which is parallel to the survey line. Quarry location is shown in Supplementary Figure S4. (**b**) Estimated shear wave velocity profile. (**c**) Histogram representation of data misfit for first and final inversion iterations along with a normal distribution fitted to last iteration misfit. (**d**) Uncertainty estimates in the inverted velocity profile. Overlay image illustrating the correspondence between the velocity model and the schematic basalt flow diagram in the inset is presented in the Supplementary Figure S12 to aid interpretation.

A total of 23 lava flows are exposed in the Katraj Ghat section^31^. A flat terrace at 1005 m amsl in the Katraj Ghat section provides an ideal location for deploying the seismic array. We established the volcano-stratigraphy at the Katraj Ghat site based on field observations in a 100 m thick lithological section exposed at the seismic survey location (refer to Supplementary Figure S6). In the exposed vertical section adjacent to the terrace (Fig. 9a), three rubbly pahoehoe lava flow layers were observed, each with a conspicuous flow-top breccia layer. In the lowest part of the exposed section, a massive basalt core is exposed, overlain by a vesicular crust.

The S-wave velocity model of the Katraj site is illustrated in Fig. 9b. The upper 4.5 m of the subsurface shows $V_S$ in the range of 1.1 to 1.4 km/s. This layer is underlain by a very low velocity ($\sim$ 0.15 m/s) layer in the 4.5-6.5 m depth range. This low-velocity layer is correlated with the flow-top breccia observed in the outcrop. The thickness of the red bole layer is found to be 2 m in the inverted model, matching its actual thickness measured in the outcrop labelled as ‘B’ in Fig. 4. This very low shear wave velocity layer likely represents the weaker shear strength of the material. A 5 m-thick layer immediately beneath this low-velocity layer, which is labelled as ‘C’ in Fig. 4, has a relatively higher velocity ( $\sim$0.5 km/s), compared to the flow-top breccia. At $\sim$11 m below the surface, the $V_S$ model shows a sharp velocity increase from 0.5 to >1.0 km/s, followed by a gradual rise to  2.0 km/s at a depth of 35 m. The S-wave velocity section shows layered characteristics, but there is a gradual reduction in velocity from the SSE to the NNW direction 20 m onward from the SSE end. Figure 9c depicts the histogram of data misfit for both the first and final sets of the $V_S$ inversion iteration. The distribution plot for the final iteration closely matches the normal distribution plot, with a standard deviation of 0.065. The uncertainties in the obtained velocity model are calculated based on the data error, which has a standard deviation of 0.065. The uncertainty plot is shown in Fig. 9d. The uncertainties in velocity are generally below 0.03 km/s except for the topmost layer, where the uncertainty is 0.07 km/s.

### Katraj (KJP2) profile

**Fig. 10:**
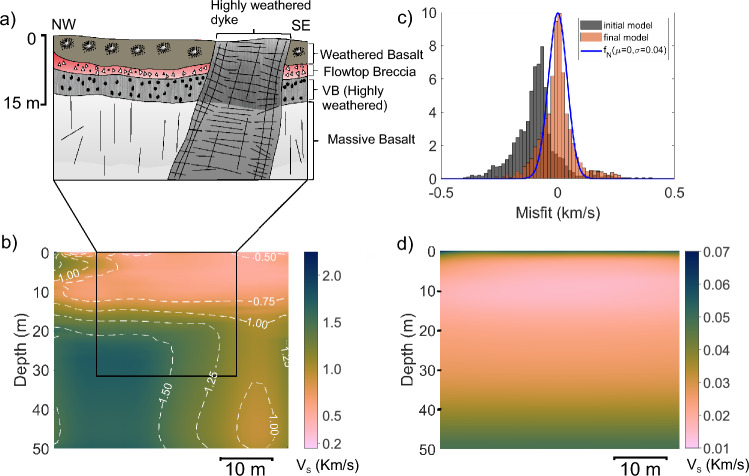
Katraj Ghat KJP2 profile results. (**a**) Schematic diagram illustrating the internal structure of lava flow in the survey area based on a section exposed in a nearby road cut which is parallel to the survey line. Road cut section location is shown in Supplementary Figure S4. (**b**) Estimated shear wave velocity profile. (**c**) Histogram representation of data misfit for first and final inversion iterations along with a normal distribution fitted to last iteration misfit. (**d**) Uncertainty estimates in the inverted velocity profile. Below 45 m depth, the model uncertainty increases, and the velocity inversion produces bending of Vs contour lines near the dyke position. However, this apparent deviation is not interpreted as a real change in dip. The dyke is expected to continue with the same dip as observed in the shallower part. Overlay image of the velocity model and lava flow layers for the inset is included in Supplementary Figure S13.

A 6.3 m-thick highly jointed basaltic dyke striking N30$\phantom{0}^o$E-S210$\phantom{0}^o$W is exposed in a valley near the Katraj Ghat old tunnel (see Fig. 1). The dyke dips 73-78$\phantom{0}^\circ$ towards NW (N60$\phantom{0}^o$W) (Fig. 10a). The dyke cuts through the flow top breccias and lava flows belonging to the Ambenali Formation^31^. The dyke shows orthogonal set of cooling joints, along-the dip of the dyke and across-dip (see Fig. 5 for field images). Due to its highly jointed nature, the dyke facilitates groundwater flow, functioning as a carrier dyke^58^.

A seismic profile oriented in the NW-SE direction, perpendicular to the dyke was acquired to image the subsurface at the Katraj Ghat site. The derived $V_S$ model, data misfit and uncertainty are shown in Fig. 10b-d, respectively. The top 13 m of the subsurface show a nearly uniform shear wave velocity ($V_S$) of 0.7 km/s, with minimal lateral variation. In the exposed vertical section, the dyke extends to the surface; however, no clear indication of its presence, such as a subtle change in velocity, is observed in the shallowest part of the section. This lack of distinction is likely due to extensive weathering, which has significantly altered the top 13 m, making the host rock and the dyke indistinguishable. Between 13 m and 20 m depth, velocity increases steadily from 0.7 km/s to 1.4 km/s. The interval between 20 and 50 m below the surface exhibits strong spatial variations in $V_S$. A lateral velocity contrast emerges at a depth of around 20 m near the midpoint of the profile and progressively dips towards the SE with increasing depth.

Field observations from the outcrop indicate that the NW-dipping interface between the dyke and the basalt flows corresponds to a prominent velocity contrast at the NW edge of the dyke in the shear wave velocity ($V_S$) model. The shear wave velocity ($V_S$) of the dyke is approximately 1.4 km/s, which matches well with the S-wave velocity recorded at the Dive Ghat site. On the SE side of the dyke, the adjacent subsurface exhibits a lower velocity ($\sim$0.8 km/s) compared to the NW side. This difference is attributed to intense chemical weathering, potentially driven by water supplied from the dyke to the adjacent basalt layers. The cooling joints, developed perpendicular to the dip direction of the dyke, facilitate gravity-driven drainage of water on the SE side.

## Discussion

**Fig. 11:**
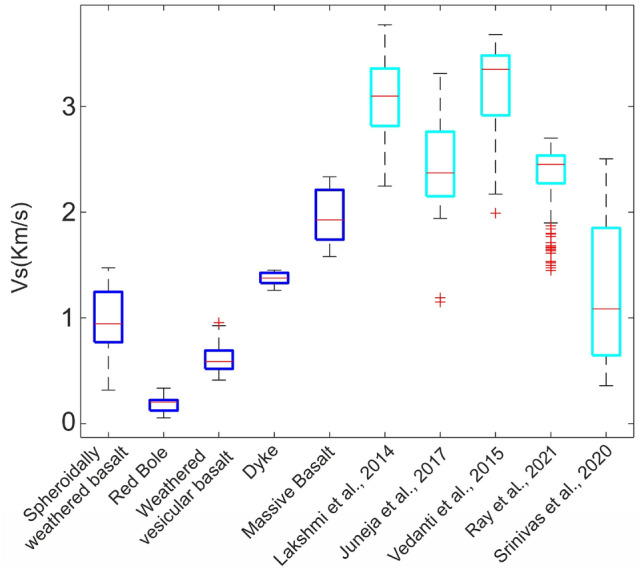
Shear wave velocity classification prepared using a co-analysis of the exposed geological section and the S-wave velocities observed in this study (blue-colored box) along with the velocities reported in the literature (cyan-colored box) from the Deccan region. Red line denotes the median velocity values. Note that velocity values used in Lakshmi et al., 2014^59^ correspond only to the Bushe and Poladpur Formations.

The shear wave velocity obtained in this study varies significantly, ranging from 0.06 km/s to 2.2 km/s. Survey sites near exposed basalt sections were strategically selected to correlate velocity values against basalt types. Consequently, the shear wave velocity comparisons were limited to the exposed sections. The velocities from subsurface models corresponding to the same basalt class were considered for comparison with other studies. Figure 11 presents a box plot of velocity ranges for different basalt facies classes in this study, alongside published data from the Deccan Volcanic Province. The velocity range varies across basalt types: spheroidally weathered basalt (0.77–1.25 km/s), bole layer (0.12–0.22 km/s), weathered vesicular basalt (0.52–0.69 km/s), dyke (1.33–1.42 km/s), and massive basalt (1.74–2.2 km/s). This plot reveals a bias toward higher shear wave velocities in lab-based sonic-log experiments^59–61^ and well-log measurements^62^. The utilization of higher frequency signals (e.g. 1 MHz in^61^ and 80 KHz in^60^) in such studies is one of the potential reasons for this bias. Additionally, laboratory studies typically analyze core or surface samples, which are often stronger and less fractured. In contrast, our velocity range aligns more closely with^63^, who also employed the MASW method. The velocity variability across basalt classes in this study serves as a valuable reference for constructing realistic petrophysical models.

### Seismic characterization of subsurface

The brecciated top of rubbly pahoehoe (red bole) shows the lowest velocity, with a median value of 0.2 km/s, and the highest velocity corresponds to the massive core units, with a median of 1.92 km/s. The massive and spheroidally weathered basalt shows a broader velocity range due to varying degrees of weathering for different profiles, consistent with field observations. The red bole layer has a relatively narrow velocity spread with a low median of 0.2 km/s. The vesicular basalt immediately below the red bole layer in Katraj and Diveghat (KJP1 and DGP1) is highly weathered and shows the second-lowest shear wave velocity; however, vesicular basalt has greater variability than the red bole layer. Dykes show least variability in velocity, with a median value of 1.37 km/s based on data from two dykes with thicknesses ranging from $\sim$2-7 m. Dykes cool slowly, allowing crystal growth similar to the core of a basalt flow and formation of numerous cooling joints, yet their velocities are substantially lower than massive basalt. This reduction is attributed to clay-filled cooling joints, which likely play a key role in lowering dyke velocity despite their small volume fraction. Secondary porosity due to joints can be estimated using a first-order approximation of joint volume within dykes, assuming that the median velocity of the lava flow core represents the dyke matrix and the median velocity of the red bole material represents the clay-filled joint volume. In accordance with rock physics principles, the porosity-weighted harmonic mean is preferred over the arithmetic mean for velocity estimation (e.g. $\frac{1}{v_s} = \frac{1 -\phi }{v_{\text {matrix}}} + \frac{\phi }{v_{\text {fluid}}}$ ,where $\phi$, $v_{\text {matrix}}$, $v_{\text {fluid}}$ denotes porosity, velocity of matrix and velocity of fluid respectively^64^), as it is sensitive to lower-velocity materials. The observed median velocity in dykes can be explained if the secondary porosity is estimated at 4.7%. The typical size of intact cubic blocks within the dyke is approximately 30 $\times$ 30 $\times$ 10 cm (Fig. 5c). Given a joint width of 2 ± 1 mm, the estimated secondary porosity is $\sim$3.3% ± 1.7%. The close agreement between these values highlights the potential of the MASW method for estimating secondary porosity, which is an important parameter in groundwater dynamics studies within Large Igneous Provinces.These findings also have implications for civil engineering applications, as discussed in Section 5.3.

### Basalt weathering profile

Determining the extent and volume of weathered zones in basaltic terrains is challenging due to the inherent variability of lava flows. Distinguishing between the effects of weathering and intrinsic lithological variations in basaltic bedrock adds to this complexity. The derived velocity models from the Dive Ghat and Katraj sites reveal a predominance of low-velocity anomalies in the top 12 m (DGP1 and KJP1, Figs. 8b and 9b). Within this upper zone, a patchy <0.5 m thick very low-velocity region (<0.2 km/s) represents soil, while a $\sim$5-9 m thick laterally continuous high-velocity zone (0.75–1 km/s) corresponds to spheroidally weathered basalt. Beneath this, the red bole layer ($\sim$0.25-2 m thick) exhibits very low velocities (0.2–0.5 km/s), followed by a second low-velocity anomaly (0.5–0.75 km/s), which characterizes weathered vesicular basalt (Fig. 11). Below this layer, velocity increases sharply to the base of the derived model. The bole layer and the weathered vesicular basalt top can be considered saprolitic, having undergone weathering both before being buried by subsequent lava flows and more recently after the overlying lava flows were denuded. The weathering pattern of basalt varies with its geological age. For instance, a $\sim$10-meter-thick weathering zone (velocity <0.5 km/s) was reported in young Hawaiian basalt, where annual precipitation ranges between 500 and 1000 mm^65^. In comparison, Pune receives an annual average rainfall of 760 mm^66^, and its older basalts exhibit well-established drainage networks, strong erosion, and steep valley walls. Since our study sites are primarily from cliffs and road sections, the soil, typically showing velocities <0.5 km/s, occurs only in patches due to erosion and limited preservation. While the $V_s$ of the red bole layer is similar to the topmost soil, its classification as a paleosol requires confirmation through geochemical and morphometric analysis^67^. Unlike granitic terrains, where shear wave velocity increases monotonically with depth, basaltic terrains exhibit more complex velocity patterns. In the Dive Ghat region, a weathering index (WI), calculated from alkali-to-alkaline-earth element ratios^41^, indicates that basalts overlying the red bole layer have higher WI values (77–40), reflecting limited weathering. In contrast, basalts below the red bole layer show lower WI values (60–28), suggesting more extensive alteration. The red bole samples, with WI values ranging from 38 to 20, indicate significant weathering of the parent basalt before being buried by subsequent lava flows. The low shear wave velocities of the red boles corroborate the higher degree of weathering reported in a previous study^41^.

### Geotechnical and civil engineering implications

The shear modulus ($\mu$) is proportional to the square of shear wave velocity ($V_s$) and linearly dependent on density ($\rho$), $\mu$ = $V_s$ $^2\rho$. Therefore, the shear modulus shows a stronger dependency on the shear wave velocity. Since the shear modulus of a rock is a critical parameter that governs its strength, rocks with low shear wave velocity are prone to slope failure. Consequently, the proposed basalt classification can aid in natural hazard investigation and contribute to resilient infrastructure development. Table 2 provides shear modulus estimates based on the median shear wave velocity and assuming a rock density in a range following the findings by^62^ from the Koyna region. Results indicate that the weathered vesicular basalt and the red bole are the weakest basalt units, with a shear modulus one and two orders of magnitude lower than the massive core, respectively. These units must, therefore, be carefully considered in infrastructure safety assessments. Since the MASW method effectively detects low-velocity anomalies, it is highly recommended to integrate such investigations into infrastructure development projects within the Large Igneous Provinces in the tropical and subtropical regions. In this study, we could not conduct time-lapse seismic experiments across wet and dry seasons to determine the maximum water saturation resulting from groundwater recharge and water table migration. As water in pore spaces further reduces shear strength, this factor should be considered into slope stability analysis.

**Table 2:** Shear modulus for various basalt types in this study. Shear modulus was derived using the median velocities shown in Fig. 11 and density values from (^62^. The uncertainties associated with density variation are included, showing that they are more than an order of magnitude smaller than the modulus value.

Basalt class	Mean density (gm/cm$\phantom{0}^3$)	Shear modulus (GPa)
Spheroidally weathered basalt	2.92 ± 0.05	2.40 ± 0.18
Red bole	2.53 ± 0.09	0.11 ± 0.004
Weathered vesicular basalt	2.65 ± 0.11	0.89 ± 0.04
Dyke	2.93 ± 0.05	5.11 ± 0.38
Massive basalt	2.93 ± 0.05	10.04 ± 0.18

### Hydrogeological controls on groundwater flow and landscape development

**Fig. 12:**
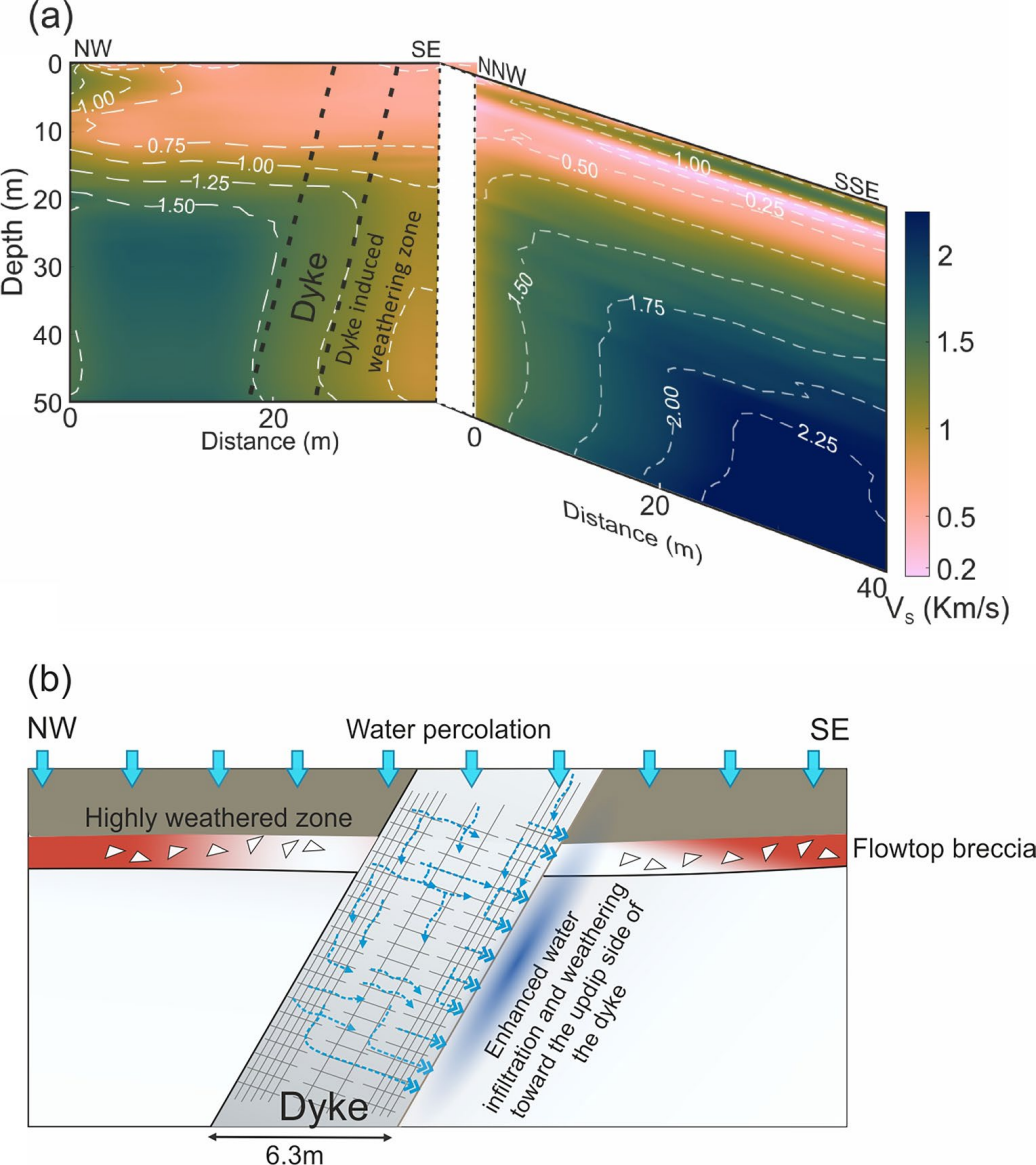
Water flow dynamics and moisture retention mechanism model for the Katraj dyke based on the subsurface velocity model. (**a**) Interpreted shear wave velocity models with the boundaries of the dipping dyke marked by the black dashed lines and low-velocity anomaly interpreted as a weathered zone on the up-dip section adjoining the dyke. (**b**) Cartoon illustrating subsurface water movement where water transmissivity is controlled by the dip of cooling joints in the dyke.

**Fig. 13:**
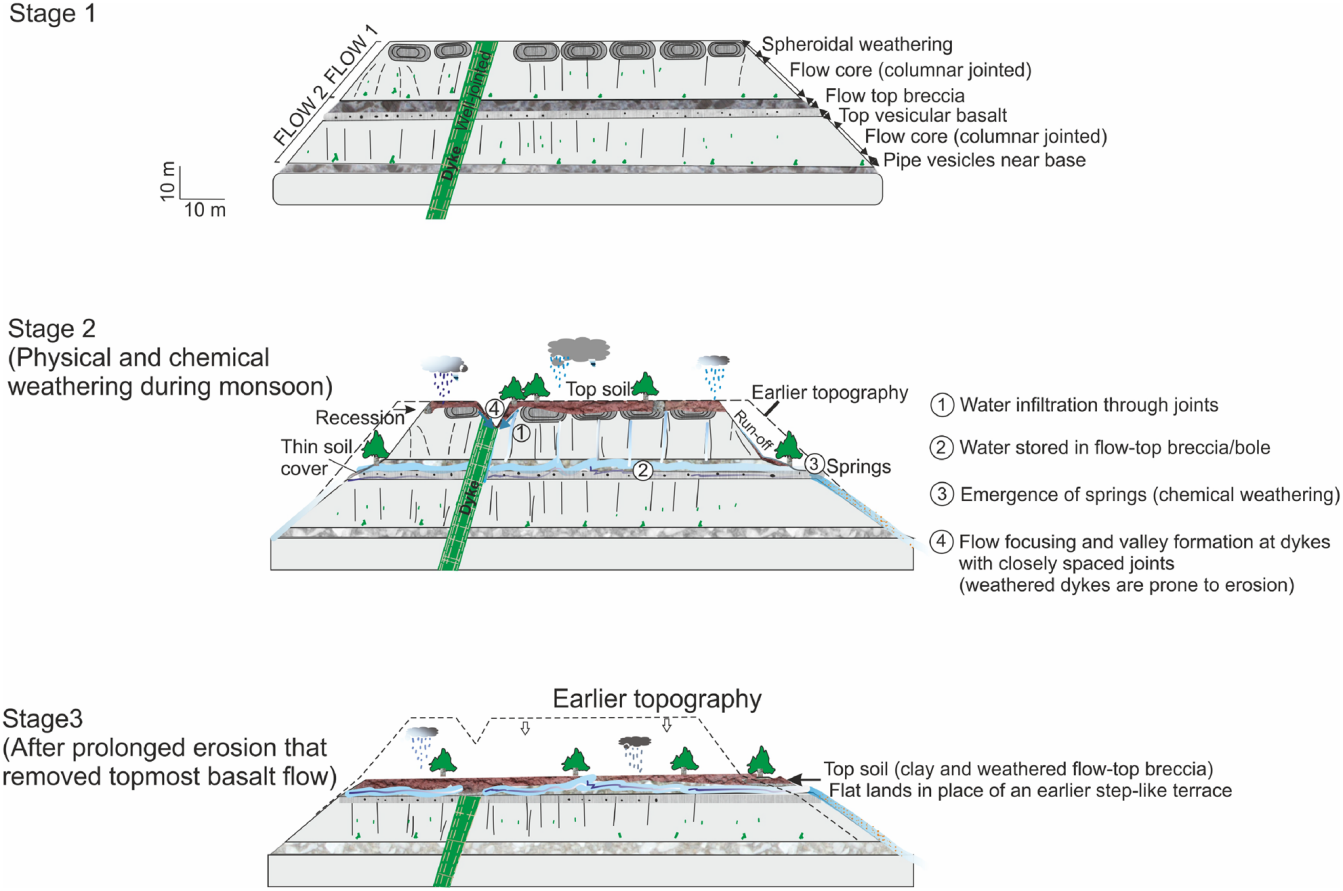
The schematic diagram illustrates key stages of landscape evolution (Stage 1 to Stage 3) in a sequence of three rubbly pahoehoe basalt flow units. Stage 1 represents the initial state, where the topmost unit consists of a massive basalt flow that has undergone spheroidal weathering. This flow overlies a red bole/flow-top breccia and weathered vesicular basalt. Stage 2 marks a slow denudation process driven by stream-led erosion and chemical weathering, primarily through hydrolysis. Water infiltrates through open joints. Weathering in dyke gets initiated near the exposed surface, and water infiltration accelerates chemical breakdown and weakens the rock. Surface runoff further deepens erosion into the dyke structure. Stage 3 begins as the fragile bole layer becomes exposed to weathering agents such as rainfall and runoff. The denudation of the bole layer is relatively rapid at this stage due to its mechanically weak and erodible nature.

**Fig. 14:**
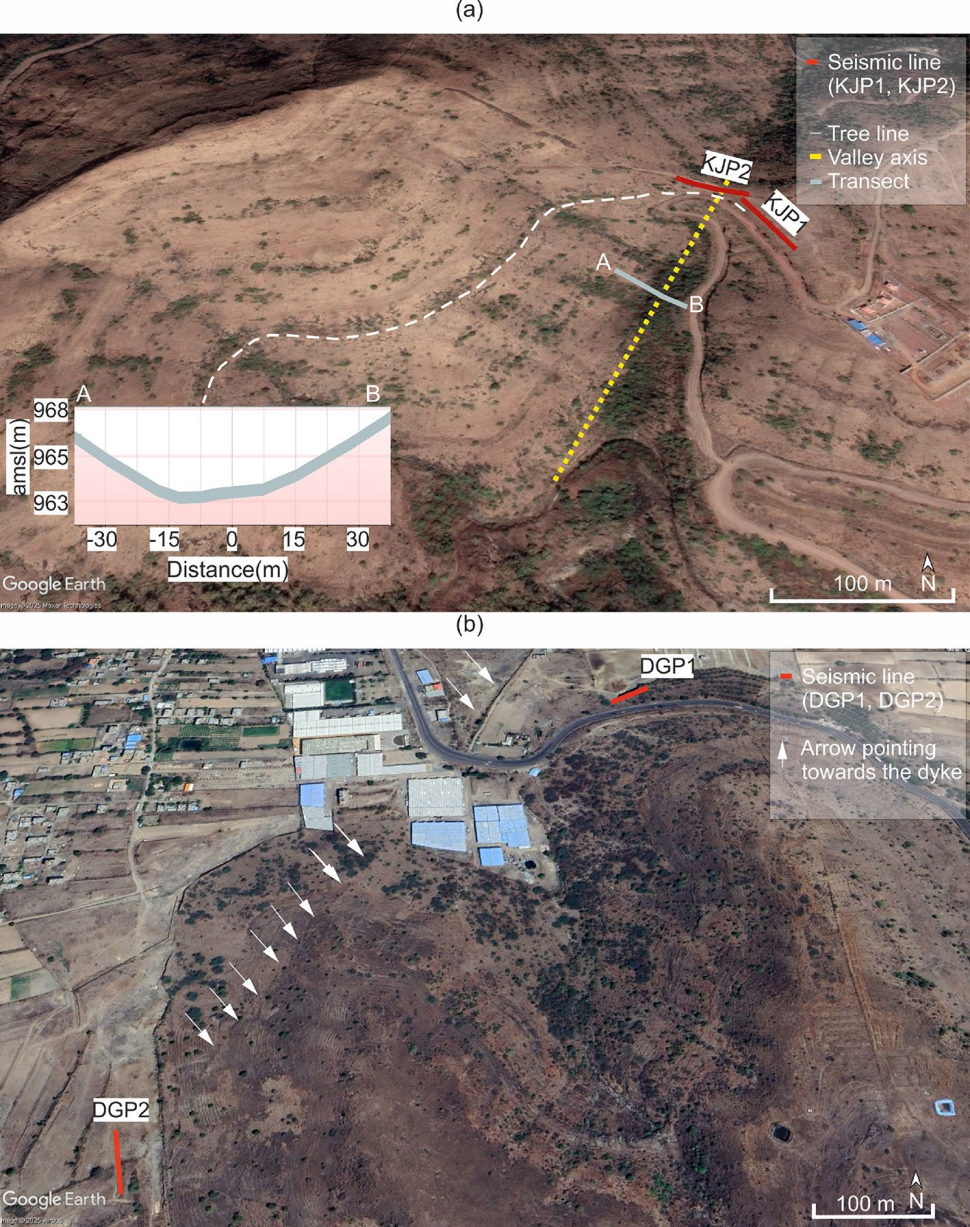
3D perspective view of Katraj and Dive Ghat study areas, where red lines represent the 2D seismic survey profiles. (**a**) Katraj study site where the dashed white line represents a tree line that follows the redbole layer; the yellow line represents the dyke passing along the valley, causing an asymmetrical development of vegetation around the axis of the valley; the elevation of the AB transect across the valley shows a symmetrical profile of the valley. (**b**) Dive ghat study site, where the white arrows point towards the signature of dyke observed in the image.

Groundwater in basaltic terrains predominantly exists in a semi-confined state within the network of fractures and joints^68^. Under a monsoonal climate regime, basaltic terrains exhibit a recharge-to-rainfall ratio of 3–15% that is dependent on precipitation variability, vegetation cover, evaporation rates, top-soil and weathered zone transmissivity and basinal topography. Water flows from the topographic drainage divides toward the central valleys. However, subsurface stratigraphic and structural complexity can further complicate the water flow paths. Groundwater flow is controlled by the highly variable hydraulic conductivity of basalt, influenced by vesicles, cooling joints, brecciated flow tops, and red boles. For instance, the bole layers trapped between two lava flows enhance lateral groundwater flow and storage potential due to their high porosity compared to hard basalt. The low-velocity vesicular basalt layer underlying the red bole is fragile. The red bole retains water for an extended period, sustaining water availability. This prolonged water retention, combined with presence of vesicles in the underlying basalt, facilitates water infiltration and enhances chemical weathering of the vesicular basalt, which can explain the lower velocity of this layer compared to the lava flow core. The shear wave velocity structure of a dipping sub-vertical dyke at the Katraj site shows a low-velocity zone along its contact with the country rock at the up-dip side. The dyke shows well-defined cooling joints perpendicular to its margins. The joint density, defined as the number of joints per unit length, is around three times higher in the cross-dip direction than the along-dip direction (Fig. 5c). The non-uniformity in joint density causes anisotropic permeability in the dyke with significantly higher permeability in the direction perpendicular to the margin. We suggest that the dip of the dyke, combined with the orientation of the dyke-perpendicular cooling joints, which likely possess higher permeability, enhances flow focusing along the dyke-lava flow contact on the up-dip side (Fig. 12). While an unjointed dyke can act as a barrier that restricts fluid movement and compartmentalizes subsurface flow regime, a highly jointed dyke located inside a valley may create localized high-transmissivity zones with open pathways, where water can infiltrate. Over time, high water flux guided by the cooling joints to one of the side walls and water retention in those wet zones can accelerate chemical weathering and transforms part of dyke and the adjoining country rock into relatively softer weathered material, which may further enhance water storage. Hence, the low-velocity zone likely signifies asymmetric weathering pattern associated with a dipping dyke. In contrast, this pattern is absent at the Dive Ghat site, where a vertical dyke likely has high transmissivity due to the vertical joints. Since the dyke cuts across a topographic ridge, it favours flow divergence and drainage (Fig. 14b).

While the observed low-velocity anomaly adjacent to the dyke is consistent with enhanced weathering along zones of increased joint density and permeability, we recognize that additional factors may contribute to the development of increased permeability and localized weathering. In particular, regional fracture sets and tectonic joint networks formed during post-emplacement deformation, as well as stress fields associated with topography, could act as preferential pathways for water infiltration and chemical alteration, either independently or in combination with the cooling joints along the dyke margins. In settings where the ratio of horizontal tectonic stress to the gravitational stress due to topographic relief is significantly less than 1, as is likely the case in our study area, the gravitational stresses dominate over horizontal tectonic compression^69^. Under these conditions, fractures beneath topographic ridges are generally expected to dip nearly vertically and strike parallel to the ridge axis. However, the low-velocity anomaly observed here is notably asymmetric and does not extend across the ridges on either side of the valley, suggesting that proximity to the dyke plays a primary role in enhancing weathering in this zone.

Landscape evolution in old (>1 Ma) basaltic terrain is primarily driven by stream erosion during peak flows from heavy precipitation, with a secondary influence from groundwater-fed springs^70^. These processes erode basalt layers, forming steeper hillslopes dissected by stream and debris flow networks (Fig. 13). Contrasting porosity and permeability at lava flow boundaries, such as the relatively high-porosity red bole and the low-porosity massive flow core, may facilitate groundwater-fed spring emergence and gradually weaken the rock through chemical weathering^71,72^. Differences in rock erodibility will influence erosion rates. Runoff may easily erode weaker layers, but a more resistant basalt cap can shield the underlying red bole from erosion. Once exposed, the bole, being highly susceptible to weathering due to its fragility, is likely to be eroded rapidly. Spheroidal weathering of the topmost basalt layer produces cubic blocks that are further separated by pressure from the roots, which accelerates the breakdown process (see the Supplementary Figure S8.). While the exact timescale of such denudation remains unknown, physical and chemical weathering will eventually remove the protective spheroidally weathered basalt, exposing the underlying fragile bole to erosional agents (Fig. 13). Landslides, as a form of catastrophic mass wasting, play a major role in accelerating denudation by removing and transporting surface material. A water-saturated bole layer with low shear strength can fail when its stability threshold is exceeded. Anthropogenic activities such as road construction and tunnelling, can further destabilize the slope, triggering slope failure. The subsurface velocity model, combined with geological sections, provides a framework for constructing a landscape evolution model (Fig. 13) that includes the aforementioned processes.

### Hydrological and geological controls on vegetation in Basaltic Terrains

Understanding vegetation variability on basalt terrains is crucial for deciphering the interplay between geologic processes, hydrology, and ecosystem dynamics. In these environments, vegetation patterns serve as indicators of underlying moisture retention, nutrient availability, and weathering processes. We elucidate the role of basalt dykes, bole layers, and weathered vesicular basalt in controlling vegetation distribution, with an emphasis on how these factors contribute to long-term water retention and chemical weathering.

The hill slope shows several tree lines at various heights with 30–50 m height intervals, which coincide with bole layers at those heights (Fig. 14a). Since these tree lines appear without significant changes in slope, their distribution is primarily controlled by the moisture provided by the bole layers and weathered basalt, with soil accumulation playing a secondary role due to the overall steepness of the slope. Tree roots on the surface extend through gaps in the spheroidally weathered basalt to reach the underlying bole layer, demonstrating an adaptive strategy to access water (refer to Figure S8 in supplementary S1.5.).

Areas adjacent to the well-jointed dykes may develop enhanced water storage, which is a critical factor for plant growth, particularly in valleys that facilitate flow convergence. At the Katraj site, satellite imagery reveals that vegetation is concentrated in a narrow region up-dip of a subvertical basalt dyke within a valley (Fig. 14a). Despite the valley’s symmetric elevation profiles, this up-dip area consistently supports vegetation throughout the dry season and summer. This observation is supported by the subsurface velocity model, which shows an asymmetric low-velocity anomaly on the up-dip side of the dyke that broadly correlates with vegetation distribution. These findings indicate that differences in water availability and retention rather than soil accumulation alone control vegetation patterns. While dykes within valleys can promote water convergence and retention, a dyke across a topographic ridge, such as a near-vertical dyke at Dive Ghat cutting across multiple basalt flows, causes flow divergence, exerting little or no preferential influence on vegetation growth along its margin (Fig. 14b).

### Broader relevance and application of MASW-based characterization in Basaltic Terrains

The MASW-based methodology applied in this study offers a rapid, cost-effective, and non-invasive means of subsurface characterization in basaltic terrains. Given the heterogeneity and complex weathering patterns typical of volcanic provinces, this approach provides critical insights into subsurface velocity structure and associated lithological variability. Below, we elaborate on the broader implications and scalability of this technique across three domains: civil engineering, volcanology, and hydrogeology.

**Civil Engineering:** In basaltic terrains, surface exposures can often be misleading due to rapid lateral and vertical facies changes, spheroidal weathering, and concealed bole layers. Accurate identification of low-velocity zones, such as bole layers and weathered vesicular basalt, is critical for infrastructure planning, foundation stability assessments, tunneling safety, and landslide hazard evaluations. The correlation between shear wave velocity and shear modulus, as demonstrated in our study, provides a direct input to geotechnical models. Integrating MASW surveys into pre-construction site investigations can aid in identifying mechanically weak zones and optimizing engineering designs, especially where conventional borehole data may be sparse.

**Volcanology:** Mapping volcanic facies in layered basalt flows is challenging due to poor exposure and lithological variations. Our velocity-based classification provides a novel geophysical proxy for identifying facies such as massive flow cores, weathered vesicular basalt, red boles, and intrusive dykes. The method can be applied to other Large Igneous Provinces (LIPs), such as the Paraná-Etendeka^73^, Karoo, Columbia River Flood Basalts^74^ and North Atlantic provinces, where thick basalt sequences with complex internal architectures are common. Given its portability and low logistical footprint, MASW is particularly suitable for remote or inaccessible areas and can complement remote sensing and stratigraphic modeling efforts to refine volcanic facies maps and eruptive histories.

**Hydrogeology:** In basaltic aquifers, permeability and groundwater movement are primarily controlled by aquifer type^75^, fractures, weathered zones, and inter-flow boundaries. Our results highlight how red bole layers and highly jointed dykes act as important controls on moisture retention and fluid pathways. The MASW-derived velocity model may enable more informed predictions of groundwater storage and recharge potential, particularly in structurally complex basaltic aquifers. The ability to resolve asymmetric weathering and structural anisotropy associated with dykes has implications for modeling groundwater-surface water interactions, spring emergence, and long-term aquifer sustainability in volcanic terrains. This methodology can thus contribute to integrated groundwater management in basaltic provinces globally. In summary, the MASW-based approach not only improves resolution in subsurface mapping of basaltic terrains but also offers scalable, multidisciplinary applications. By placing the technique within broader geological, engineering, and hydrological contexts, our study provides a transferable framework that can aid researchers and engineers across various volcanic landscapes worldwide.

## Conclusion

We present the first detailed S-wave velocity characterization of the uppermost 50 m of basalt lava flows exposed around Pune in the Deccan Volcanic Province and provide new seismic velocity constraints on subsurface heterogeneities in these flows using the MASW method. Our results, validated against outcrop observations^76,77^, distinguish key basalt facies, including spheroidally weathered basalt, massive basalt forming the flow core, highly weathered vesicular basalt, dykes, and red bole layers. While previous studies (e.g.,^63^) provided broad velocity ranges for different basalt facies, our findings refine these estimates and provide new insight into their mechanical and hydrological significance.

The highly weathered vesicular basalt and fragile red bole layers, with their high porosity, play a critical role in groundwater storage. Among all facies, red bole layers exhibit the lowest seismic velocities and may be indistinguishable from soil. Their weak mechanical properties and low shear strength make them particularly prone to shear failure compared to other basalt facies.

We identify near-vertical dykes with S-wave velocities of $\sim$1.4 km/s at Dive Ghat and Katraj Ghat. While most basalt flow layers are horizontal, our seismic velocity inversion confirms the presence of high-angle dykes that are discordant to the lava flow stratigraphy. Due to the resolution limitations of the MASW method at greater depths, thickness estimates for red bole layers are more reliable in the shallowest sections at Katraj Ghat. However, multiple deeper red bole layers extend beyond the penetration depth of our seismic data and could not be imaged. These findings highlight the capability of the MASW method to resolve complex volcanic stratigraphy and structures and have direct implications for groundwater management, slope stability, and engineering applications in basaltic terrains.

## Supplementary Information

Supplementary Information.

## Data Availability

Data can be made available upon request from the corresponding authors.
