# Supplementary material for: Seismic characterization of lava flow facies in the critical zone of the deccan traps using shear wave velocity models
Source: Sci Rep. 2025 Aug 1;15:28091. doi: 10.1038/s41598-025-13638-4 (PMC12316932; doi:10.1038/s41598-025-13638-4)
Supplement: Supplementary file 1 — Supplementary Information. [file 41598_2025_13638_MOESM1_ESM.pdf]

# Supplementary Material for:
Seismic Characterization of Lava Flow Facies in the Critical Zone of the Deccan Traps Using Shear Wave Velocity Models

Rashi Sharma; Rahul Dehiya; Sudipta Sarkar; Raymond Duraiswami

## S1.1. Basalt lava flow facies

This section provides a brief physical description of the different lava flow facies observed during our fieldwork in the Pune region. The descriptions and genetic characterizations provide the background information for understanding the lava flow stratigraphy. The lava flow morphology is influenced by its composition, viscosity, temperature during emplacement, and the effusion rate. Viscosity, in particular, depends on factors like vapor loss and chemical differentiation.

1. **Sheet Pahoehoe**

   **Characteristics:** Sheet pahoehoe is a type of pahoehoe lava flow that is characterized by laterally extensive, and continuous lava sheets. This structure consists of three distinct units-the vesicular crust, the flow core, and a basal pipe-bearing zone through which gas escapes from a solidifying lava. The core often shows columnar jointing.

   **Genetic Significance of the observed Features:** Pahoehoe lava forms when low-viscosity basaltic lava spreads out over a broad area and creates a relatively flat and nearly uniform surface. The flows show distinct inflation of lobes that form as flat-topped sheets. The vesicular crust forms due to gas release from the top as lava cools in contact with the atmosphere. The flow core remains molten for a longer duration and allows lava movement. Columnar joints that are approximately perpendicular to flow boundaries, along with regularly spaced horizontal joints, form during the cooling of the lava flow. These three-dimensional joint patterns divide the rock into blocks. These blocks become susceptible to spheroidal weathering. Spheroidal weathering is a surface weathering process commonly observed in basalt with pre-existing blocky joints. The outer layers or rinds of these blocks are weakened over time, eventually causing them to detach through exfoliation. This process results in the characteristic rounded shapes often seen in weathered basalt.

2. **Rubbly Pahoehoe**

   **Characteristics:** Rubbly pahoehoe lava flows are characterized by intact bases and fragmented and brecciated upper crusts. It results from slightly higher lava effusion rates compared to typical pahoehoe flows. The rubbly top is also referred to as flow top breccia (FTB) due to faster cooling, hardening and fragmentation.

   **Genetic Significance of the observed Features:** This type of lava flow represents a transition between pahoehoe and 'a'ā type of flows, and form under slightly higher effusion rates than pahoehoe. The increased lava discharge influences the surface texture and flow dynamics, and leads to intermediate characteristics between the two end-member morphologies.

3. **Vesicular/Amygdular basalt**

   **Characteristics:** This basalt lava flow type is characterized by the presence of vesicles, which are escape routes of gas bubbles trapped during lava solidification. In some cases, these vesicles are later filled with secondary minerals, forming amygdules.

   **Genetic significance:** The vesicles form due to gas release as the lava cools down and solidifies. The varying sizes and shapes of vesicles indicate differences in gas content and cooling rates. Highly deformed vesicles suggest degassing occurred under shear deformation during lava flow. The presence of amygdules indicates late-stage mineralization due to fluid infiltration into the vesicles.

4. **Red Bole layer**

   **Characteristics:** Red bole is a brick-red coloured, fine-grained, clay-rich layer found between volcanic flows. It often shows signs of induration due to the baking effect of an overlying basalt flow. In some cases, it contains brecciated material with clasts of variable sizes. They may also show signs of spheroidal weathering.

   **Genetic Significance:** Red bole formed during periods of volcanic quiescence, during which weathering and alteration of volcanic material occur. Spheroidal weathering indicates chemical alteration processes, while the presence of the brecciated rubble with diverse clast sizes, including ball-shaped clasts, suggests that the lava mechanically picked up and entrained surface fragments.

5. **Dykes**

   **Characteristics:** A dyke is a sheet-like igneous intrusion that cuts across pre-existing rock layers, making it discordant to the surrounding country rocks. These structures are commonly composed of mafic rocks, such as basalt or dolerite, which contain minerals like plagioclase, pyroxene, and olivine. Dykes vary in thickness, ranging from a few centimeters to several meters, and can extend over long distances, sometimes forming extensive dyke swarms.

   **Genetic Significance:** Dykes form when magma migrates through fractures in the Earth's crust and solidifies underground before reaching the surface. As the magma comes into contact with cooler country rocks, it cools rapidly at the edges, forming a fine-grained texture known as the chilled margin. The process of magma cooling also leads to contraction, which results in the formation of cooling joints, typically oriented perpendicular to the dyke walls. These structures are important

in understanding tectonic and magmatic processes, as they often serve as conduits for magma transport.

## S1.2. Quarry Locations near the Seismic lines

Quarry locations present around the 2D seismic lines are shown in the Figures S1 - S4. Objective of these images is to show the location of quarry/outcrop with respect to Seismic profile where field photographs are taken for validation purpose. Field photographs are kept at actual vertical scale and are not vertically exaggerated.

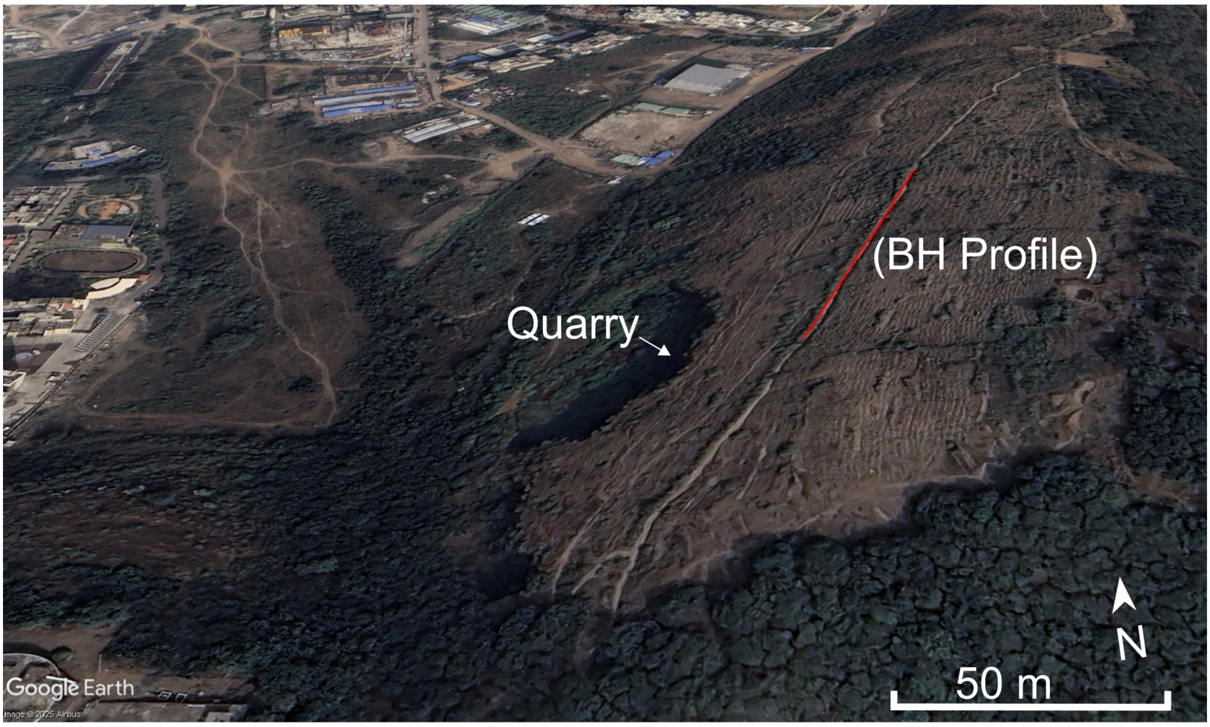


**Figure S1:** Baner hill quarry present on the west side of 2D seismic line shown in red color.

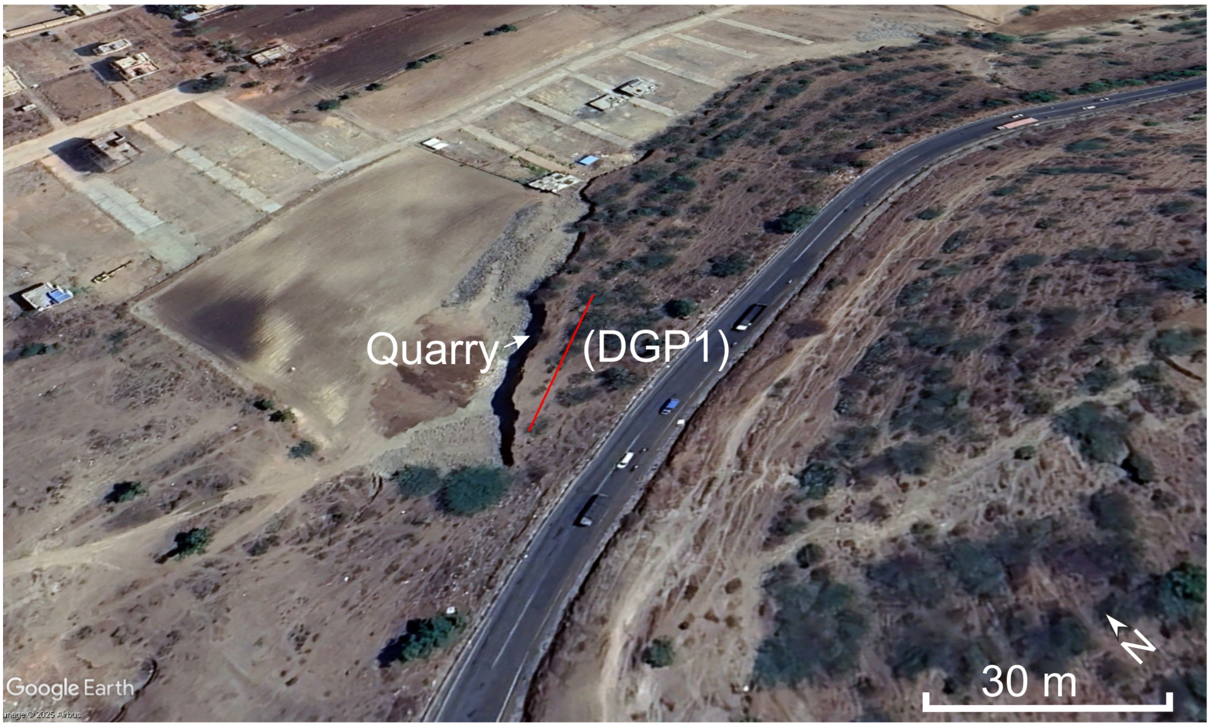


**Figure S2:** Dive ghat location showing 2D seismic line indicated by red color, labelled as DGP1 and a quarry present just next to it.

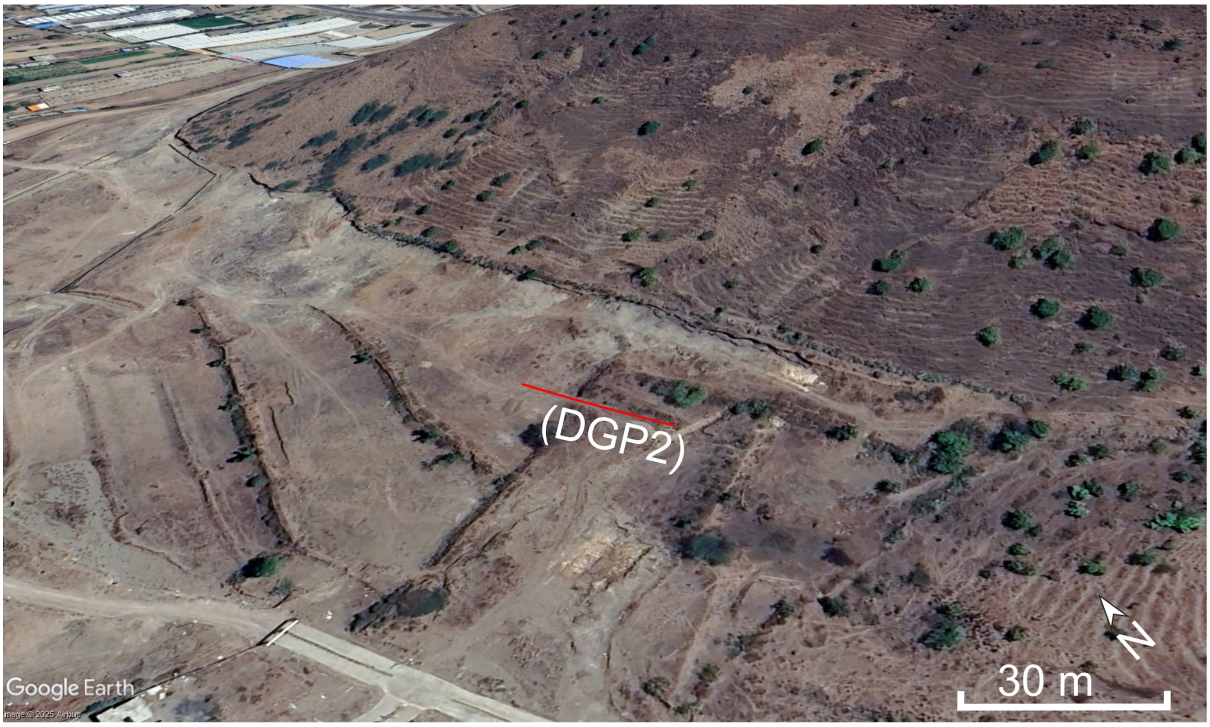


**Figure S3:** Seismic data acquired along red line labelled as DGP2 and nearby outcrop present in the field.

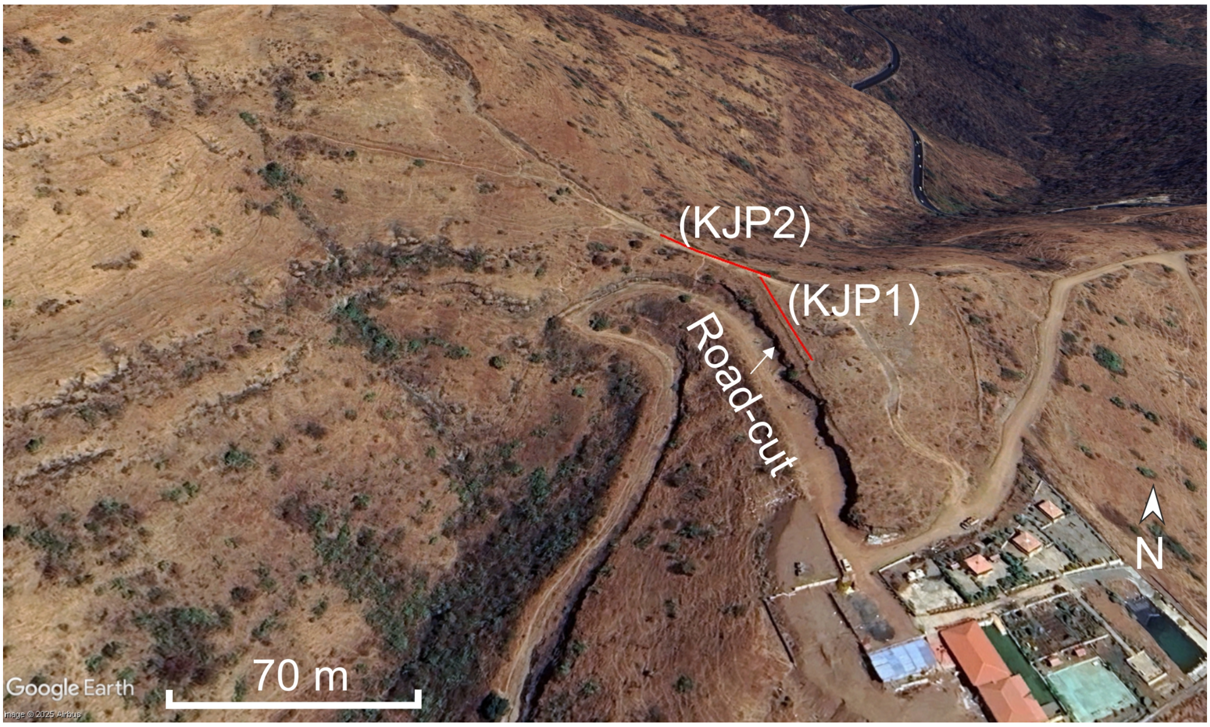


**Figure S4:** Katraj ghat location showing the 2D Seismic lines KJP1 and KJP2 with red color along with the road-cut exposed nearby it.

# S1.3. Petrographic analysis of basalt flows

The samples from the dykes and lava flows were collected and subjected to petrographic studies. All dykes and flows are olivine-bearing tholeiite basalts. Most dykes are porphyritic with plagioclase phenocrysts ranging from 0.5 to 1 cm. Using the dollar modal counting stage the modal analysis of the lava flows and the dykes are presented in Table S1. The plagioclase microphenocrysts constitute 27 - 43 volume% while the groundmass plagioclases varied from 2 -10 volume%. In thin sections plagioclase typically occurs as lath shaped microphenocryst. At places they form typical glomeroporphyritic aggregates (Figure S5(a)). In Dive Ghat lava flow, the plagioclase and clinopyroxene microphenocrysts are present in a fine grained-groundmass. In the Katraj Ghat lava flow, the plagioclase microphenocrysts occurs along with olivine (Figure S5(b)). The olivine content in the lava flows varies from 0.2 to 1.5 volume%, while the clinopyroxenes varies from 31 to 40.5 volume%. Coarse grained texture is observed in dyke rocks from the study area. The Dive Ghat dyke shows typical porphyritic texture but the groundmass is characterized by sub-ophitic texture (Figure S5(c)). In the Katraj dyke the microporphyritic plagioclase is a typical texture (Figure S5(d)). Devitrified glass occurs as 6 to 25 volume percent in the studied lava flows and to a lesser extent in the dykes.

| Location | Form | Sample Number | Plg (g) | Plg(p) | Cpx | Ol | Opg | Glass |
|---|---|---|---|---|---|---|---|---|
| Katraj Ghat | Flow | F18 | 30.17 | 3.50 | 31.10 | 0.20 | 10.0 | 25.03 |
| | Flow | F17 | 31.17 | 8.38 | 40.46 | 0.40 | 10.07 | 9.28 |
| | Dyke | PK2 | 43.20 | 3.14 | 32.65 | – | 11.23 | 9.78 |
| Dive Ghat | Flow | F7 | 27.57 | 7.20 | 32.14 | 1.50 | 11.4 | 20.19 |
| | Flow | F6 | 42.73 | 2.56 | 38.46 | 0.85 | 8.54 | 6.86 |
| | Dyke | PD1 | 37.14 | 5.46 | 34.82 | 1.16 | 10.52 | |
| Sus Road | Flow | F4 | 35.10 | 10.40 | 33.36 | 1.05 | 12.16 | 7.91 |

**Table S1:** Modal (Volume%) of minerals and glass in lava flows and dykes from the present study. Note that the value in the box indicated by "-" means that the mineral is not present in the sample.

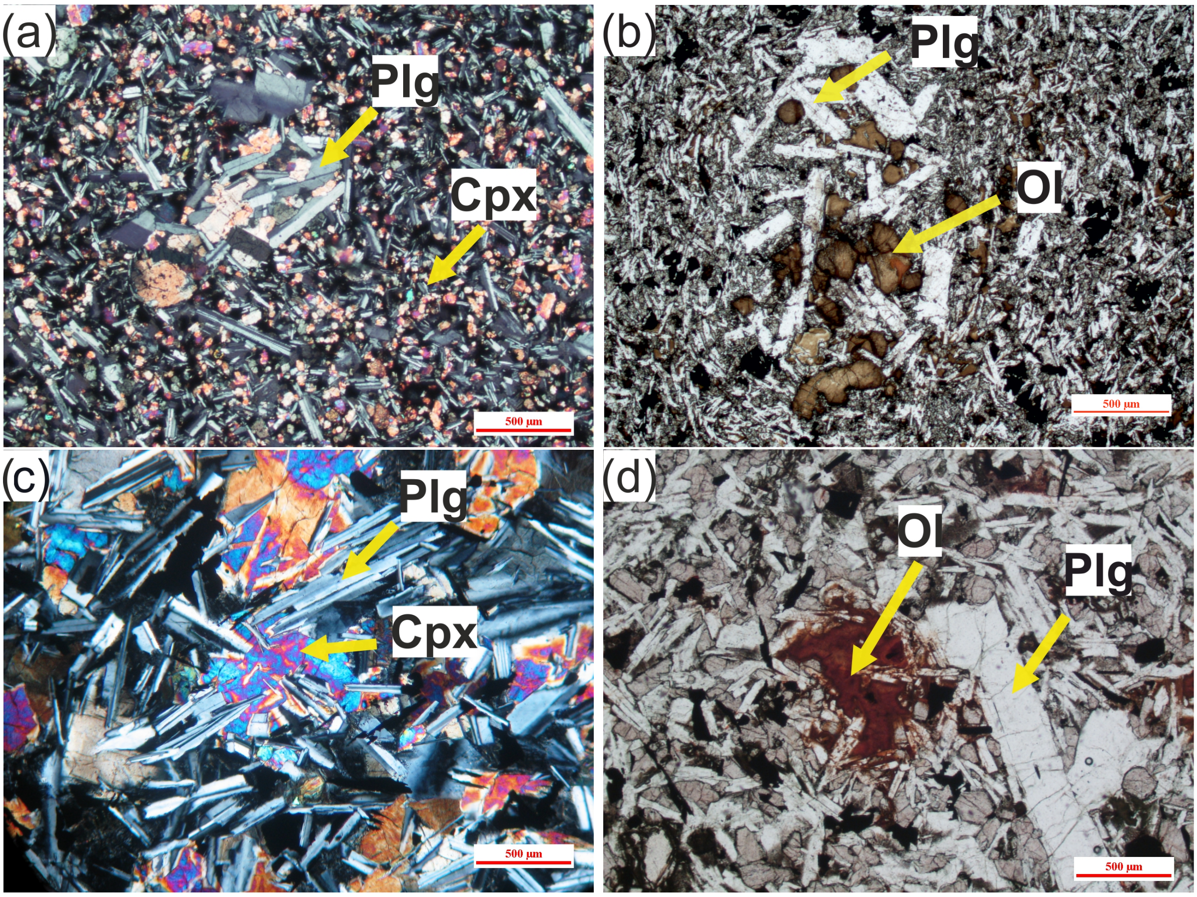


**Figure S5:** Photomicrographs of (a) lava flow exposed at Dive Ghat showing glomeroporphyritic texture by plagioclase and pyroxenes in a fine-grained groundmass. (b) Olivine-plagioclase aggregate in the Katraj Ghat lava flow. (c) Sub-ophitic texture shown by plagioclase-pyroxene in the Dive Ghat dyke. (d) Plagioclase micro phenocrysts and glass patches in a medium grain groundmass as seen in the Katraj Ghat dyke.

## S1.4. Lithological column at Katraj Ghat

The schematic representation of a lithological column (Figure S6) is developed based on the exposed section along 100 m walking track beginning at a roadside up to the survey site.

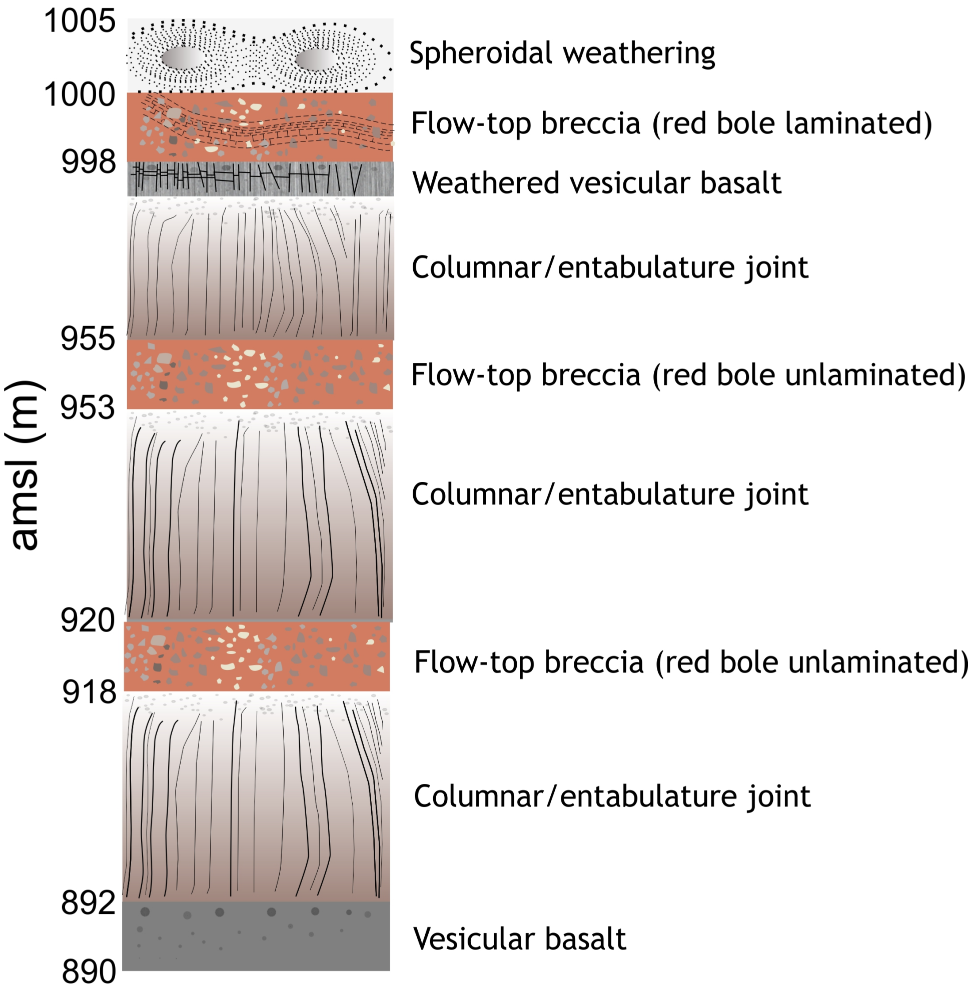


**Figure S6:** Schematic representation of basalt flows at the Katraj Ghat site present along the track just below the seismic survey line. An approximately 2 m-thick flow-top breccia/red bole layer separates two successive flow units.

## S1.5. Examples of field photographs from Baner Hill and Katraj Ghat sites

Geological fieldwork was carried out near the seismic survey area to build the understanding of the geological features present in the area. The geological features visible at the exposed section are illustrated using the field photograph at various scale as shown in Figures S7 and S8.

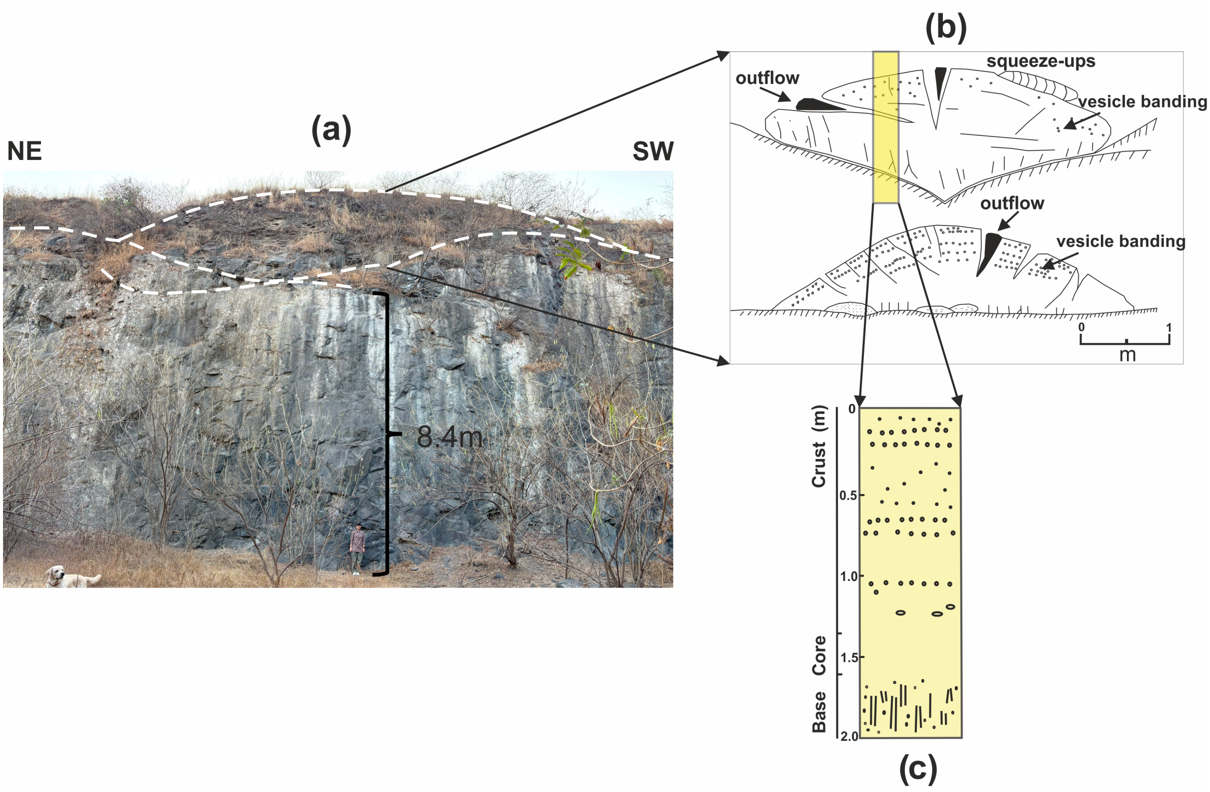


**Figure S7:** A cross-section view of the Baner hill site. (a) Section showing hummocky lobes. Inflation structures such as tumuli develop due to internal pressure variations and lava squeeze-ups as it extrudes through fractures in the hardened crust. (b) A schematic representation of tumuli showing the squeeze-ups, outflow and vesicle banding [54]. (c) Vesicle distribution profile along with the section observed in the field with large vesicles of size 1.78 cm seen at 1 m below the surface [54].

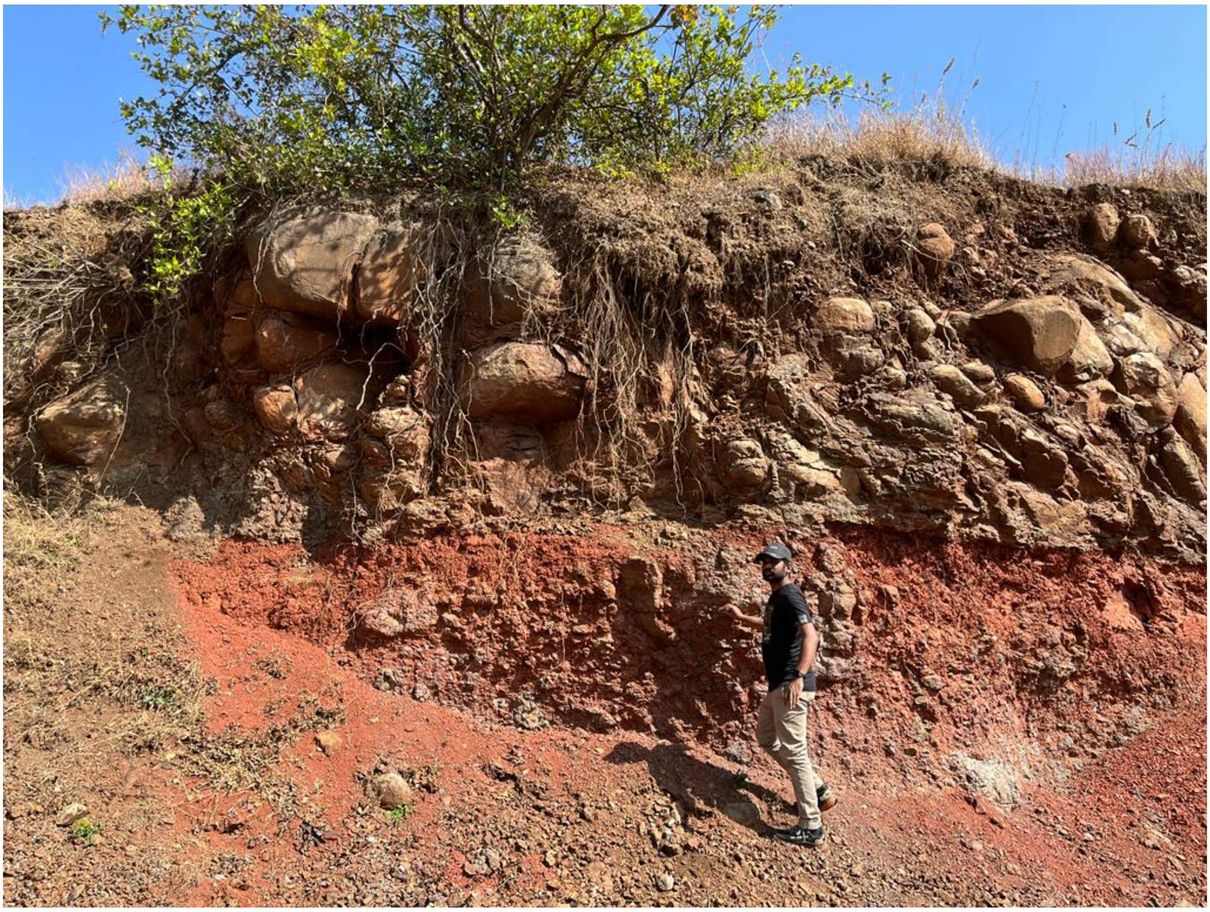

**Figure S8:** Tree roots at Katraj site are seen penetrating through joints in spheroidally weathered basalt and reach the bole layer.

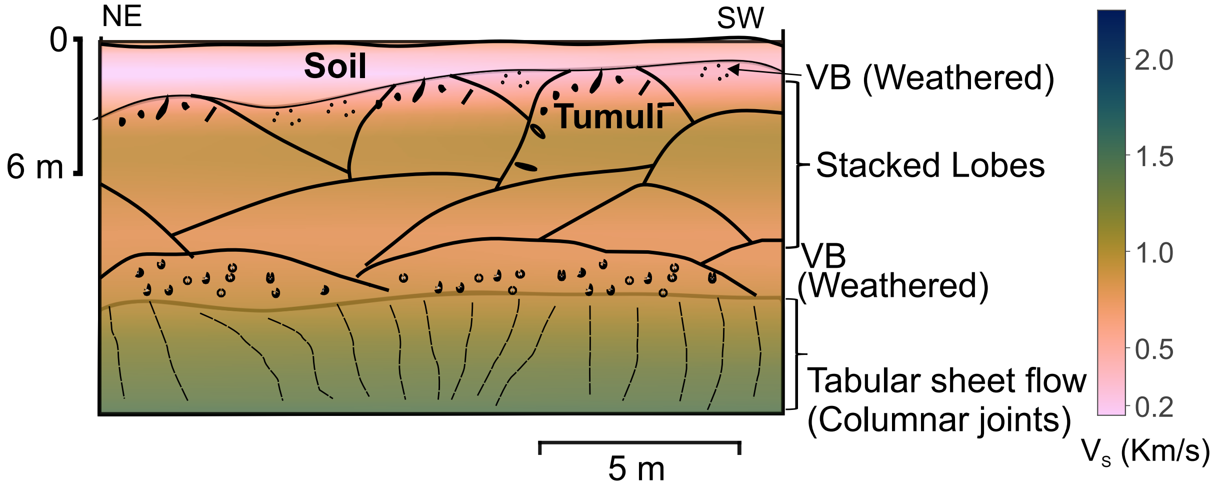


**Figure S9:** Seismic velocities overlaid on the schematic representation of outcrop at baner hill quarry.

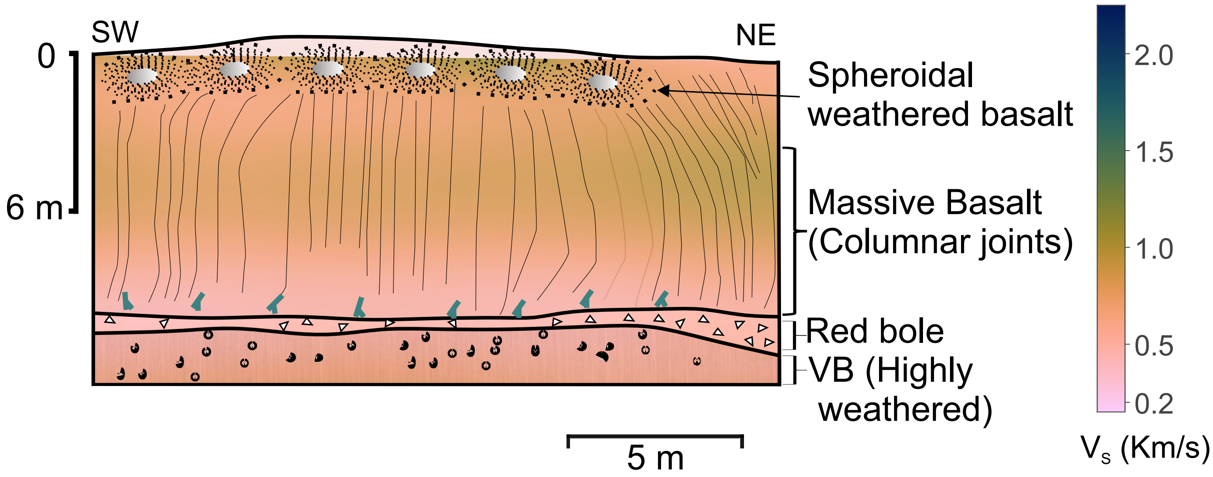


**Figure S10:** Seismic velocities overlaid on the schematic representation of outcrop at Dive ghat outcrop.

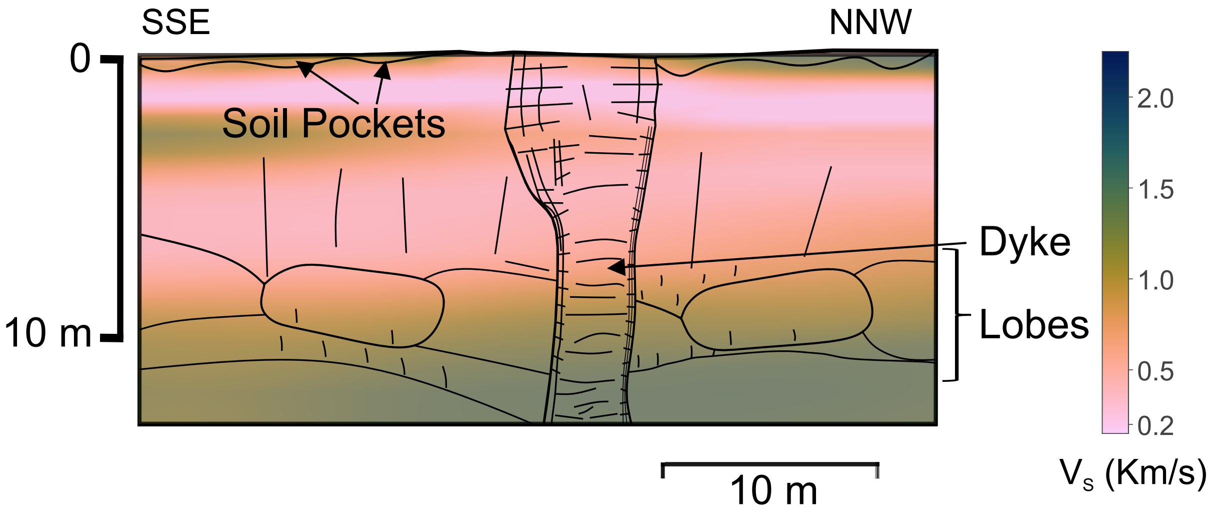


**Figure S11:** Seismic velocities overlaid on the schematic representation of nearby outcrop at Dive ghat dyke.

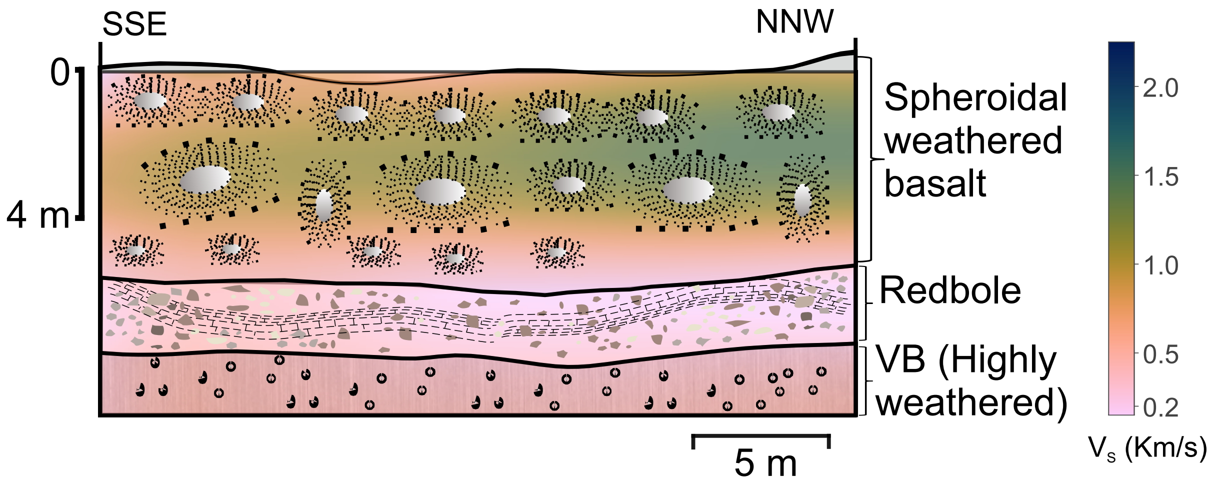


**Figure S12:** Seismic velocities overlaid on the schematic representation of outcrop at Katraj quarry having dyke.

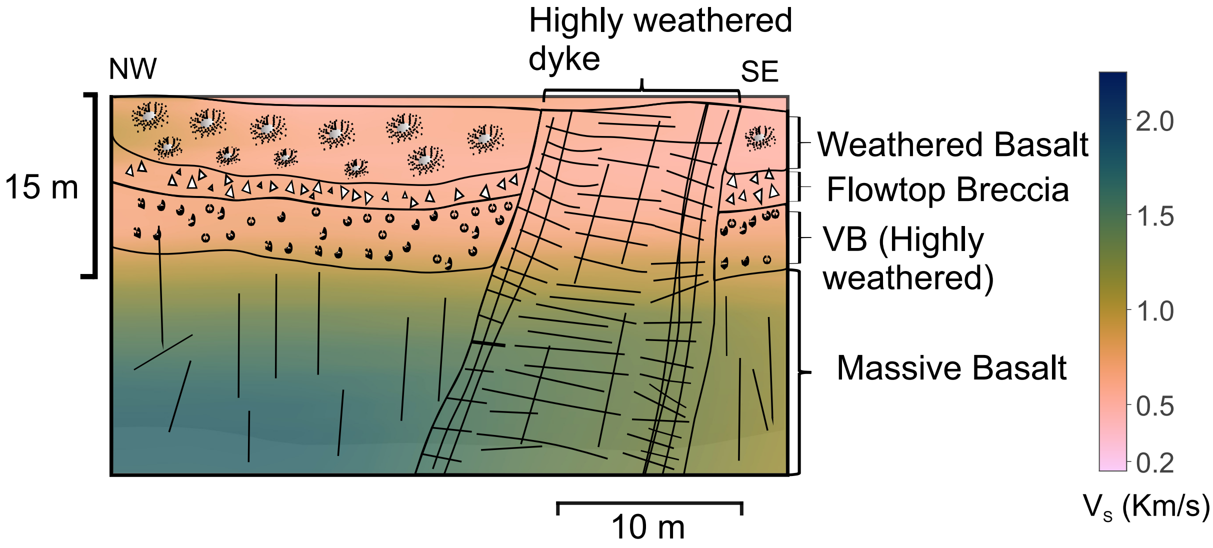


**Figure S13:** Seismic velocities overlaid on the schematic representation of outcrop at Katraj quarry having bole layer.
